# Quantitative systems pharmacology model of erythropoiesis to simulate therapies targeting anemia due to chronic kidney disease

**DOI:** 10.3389/fphar.2023.1274490

**Published:** 2023-12-06

**Authors:** Mrittika Roy, Shaifali Saroha, Uddipan Sarma, Harini Sarathy, Rukmini Kumar

**Affiliations:** ^1^ Vantage Research Inc., Lewes, DE, United States; ^2^ Division of Nephrology, University of California San Francisco, Zuckerberg San Francisco General Hospital, San Francisco, CA, United States

**Keywords:** erythropoiesis, anemia, chronic kidney disease, erythropoiesis-stimulating agents, prolyl hydroxylase inhibitors, quantitative systems pharmacology, clinical trial simulation, virtual population

## Abstract

Anemia induced by chronic kidney disease (CKD) has multiple underlying mechanistic causes and generally worsens as CKD progresses. Erythropoietin (EPO) is a key endogenous protein which increases the number of erythrocyte progenitors that mature into red blood cells that carry hemoglobin (Hb). Recombinant human erythropoietin (rHuEPO) in its native and re-engineered forms is used as a therapeutic to alleviate CKD-induced anemia by stimulating erythropoiesis. However, due to safety risks associated with erythropoiesis-stimulating agents (ESAs), a new class of drugs, prolyl hydroxylase inhibitors (PHIs), has been developed. Instead of administering exogenous EPO, PHIs facilitate the accumulation of HIF-α, which results in the increased production of endogenous EPO. Clinical trials for ESAs and PHIs generally involve balancing decisions related to safety and efficacy by carefully evaluating the criteria for patient selection and adaptive trial design. To enable such decisions, we developed a quantitative systems pharmacology (QSP) model of erythropoiesis which captures key aspects of physiology and its disruption in CKD. Furthermore, CKD virtual populations of varying severities were developed, calibrated, and validated against public data. Such a model can be used to simulate alternative trial protocols while designing phase 3 clinical trials, as well as an asset for reverse translation in understanding emerging clinical data.

## 1 Introduction

In this paper, we first lay out the motivation behind addressing chronic kidney disease (CKD)-induced anemia, the current standard of care, and novel therapies currently in development. We chose to capture phase 2/phase 3 clinical trials using a quantitative systems pharmacology (QSP) modeling approach, accounting for the relevant physiology and developing a virtual population (Vpop) that can be used to simulate such trials. Vpop is created by developing virtual patients (VPs) of increasing disease severity (healthy, CKD stages 3–5). From such a set of VPs, a subset of VPs which are consistent with non-dialysis (ND) and hemodialysis (HD) populations are generated to match clinical trial inclusion criteria. In particular, we also show that the QSP approach tackles adaptive trial design and can be used to visualize individual patient trajectories as their particular dose regimen and hemoglobin (Hb) response can be highly variable. We conclude with the potential applications of this work in drug development and personalized medicine and also discuss the limitations and next steps.

## 2 Background

### 2.1 Anemia in CKD

CKD refers to cumulative pathological changes in the kidneys that result in progressive decreases in kidney function that can ultimately lead to kidney failure and end-stage renal disease (ESRD) (Coresh et al., 2007; KDIGO, 2013). This disorder is viewed as a global public health concern with the increasing incidence of CKD-associated comorbidities worldwide, including diabetes, hypertension, and obesity (Bikbov et al., 2020). The Kidney Disease: Improving Global Outcomes (KDIGO) organization defines CKD based on persistent abnormality of kidney function (based on the glomerular filtration rate [GFR]) or structural damage (albuminuria). The KDIGO CKD staging uses GFR and albuminuria to categorize CKD into five clinical stages, with more severe stages corresponding to lower GFR (for example, CKD-G4 has eGFR 16–29 mL/min/1.73 m^2^; CKD-G5 or kidney failure has eGFR<15 mL/min/1.73 m^2^) (Stauffer and Fan, 2014; Levey et al., 2020).

According to the WHO, anemia is defined as Hb levels <12.0 g/dL in women and <13.0 g/dL in men (Cappellini and Motta, 2015). The prevalence of anemia or deficiency of RBCs is nearly double in individuals with CKD compared to the general population and more common among those with diabetes than those without diabetes at any level of GFR (El-Achkar et al., 2005; O'Mara, 2008; Stauffer and Fan, 2014; Inker et al., 2019). Among patients with CKD, the prevalence of anemia increases with advancing stages of CKD, i.e., lower levels of GFR. For instance, analyses of the United States-based National Health and Nutrition Examination Survey (NHANES) data combined from 2007–08 and 2009–10 cohorts showed that the prevalence of anemia increased from 8.4% in CKD Stage 1 (eGFR ≥90 mL/min/1.73 m^2) to 50.3% in CKD Stage 4 (eGFR 15–29 mL/min/1.73 m^2) and 53.4% in CKD Stage 5 (eGFR <15 mL/min/1.73 m^2) (Stauffer and Fan, 2014).

In CKD, anemia results primarily from a relative deficiency in erythropoietin (EPO) production in the kidney that progressively worsens with lower GFR (Babitt and Lin, 2012). However, other factors, such as shortened RBC lifespan, EPO resistance due to local or systemic inflammation, diabetes, absolute iron deficiency, especially due to gastrointestinal bleeding or impaired absorption of iron, functional iron deficiency due to hepcidin-induced disordered iron metabolism, and reduced level of vitamin B12 or folic acid, can also contribute significantly to anemia (Hsu et al., 2002; El-Achkar et al., 2005; de Francisco et al., 2009; Yilmaz et al., 2011; Babitt and Lin, 2012; Koury and Haase, 2015).

In individuals with CKD, anemia is associated with faster CKD progression (Mohanram et al., 2004; Portolés et al., 2013) as well as increased cardiovascular risk, hospitalizations, and all-cause mortality (Go et al., 2004; Go et al., 2006; Minutolo et al., 2022). Studies show that proper management of anemia in CKD is associated with improved symptoms and quality of life, along with reduced need for transfusion and a reduced risk for hospitalization (Pisoni et al., 2004; Schmidt and Dalton, 2007). In dialysis patients, partial correction of anemia to an Hb level <11 g/dL reduces cardiovascular mortality and hospitalizations (Besarab et al., 1998; Young et al., 2023). On the other hand, the CREATE trial concludes that early complete correction of anemia in CKD Stage 4 and 5 patients through epoetin beta administration does not reduce the risk of cardiovascular events (Eckardt, 2001; Drüeke et al., 2006).

### 2.2 Current treatments for CKD-related anemia and limitations

Treatment options for anemia due to CKD currently include blood transfusion, oral or intravenous iron supplementation, and Erythropoiesis-stimulating agents (ESAs). Blood transfusions are associated with several adverse effects, such as thromboembolic events, volume overload, suppression of the immune system, increased infection risk, iron overload, and sensitization to human leukocyte antigens that increases the risk of rejection in kidney transplant patients (Slotki, 2005; Krapf and Hulter, 2009; Macdougall and Obrador, 2013; Scornik et al., 2013). For these reasons, the use of transfusions is limited in severe anemia. Iron supplementation, especially intravenous iron supplementation, is also associated with iron overload, infection risk, and oxidative stress (Macdougall et al., 2016).

ESAs, such as recombinant human EPO (epoetin alfa or rHuEPO) and its hyperglycosylated derivative darbepoetin alfa, are standard-of-care therapies for the treatment of anemia associated with CKD. While rHuEPO has almost the same half-life and biological activity as endogenous EPO, darbepoetin alfa is long-acting with a half-life 3–4 times longer than that of rHuEPO (Elliott et al., 2008). The increased half-life of darbepoetin alfa has been proven to be beneficial to patients in terms of having a convenient dosing interval and thereby improving compliance.

However, higher Hb excursions caused by ESAs have been associated with the increased risk of cardiovascular events (Singh et al., 2006; Krapf and Hulter, 2009; McCullough et al., 2013; Scornik et al., 2013). Specifically, an increased risk for Cardiovascular disease related hospitalizations was observed in the Correction of Hemoglobin and Outcomes in Renal Insufficiency (CHOIR) trial of patients with CKD, while an increased risk of stroke was observed in the Trial to Reduce Cardiovascular Events with Aranesp (darbepoetin) Therapy (TREAT) in patients with CKD receiving kidney replacement therapy (KRT) or dialysis. The *post hoc* analysis of CHOIR hypothesized that toxicities related to high-dose rHuEPO could result in poorer outcomes among subjects with higher Hb targets (Hb >13 g/dl), particularly if they did not achieve their target Hb level. Patients unable to reach the target Hb level despite the administration of the highest doses of epoetin alfa (>10,095 IU/week) had the highest risk of cardiovascular events (Singh et al., 2006). The concerns regarding an increased risk of cardiovascular events with high dose of rHuEPO have led to a marked decrease in both rHuEPO dosing and mean Hb levels in the US dialysis populations. This also coincided with an initial increase in the prevalence among US patients on KRT/dialysis who received at least one blood transfusion in a year, followed by a decrease in transfusions (22.5% in 2019) (McMahon and Singh, 2019; United States Renal Data System, 2022). However, a recent meta-analysis suggests that the comparative effects of different ESAs on blood transfusions, death (any cause and cardiovascular), major cardiovascular events, myocardial infarction, stroke, vascular access thrombosis, kidney failure, fatigue, and breathlessness remain uncertain (Chung et al., 2023).

### 2.3 Prolyl hydroxylase inhibitors

A novel class of drugs known as hypoxia-inducible factor–prolyl hydroxylase (PHD) inhibitors (HIF-PHIs, or PHIs in short) is currently in development for the treatment of anemia in CKD. Prolyl hydroxylase inhibitors are a class of orally administered, small-molecule drugs that inhibit PHD enzyme activity. PHD enzymes induce rapid proteasomal degradation of the HIF transcription factor. They act through competitive inhibition of 2-oxoglutarate, which is essential for the hydroxylation of proline in HIF-α subunits. Inhibiting PHD enzymes thus leads to reduced HIF-α degradation. This leads to increased HIF-α accumulation, which increases HIF-mediated transcription of the major target gene *EPO*.

**FIGURE 1:**
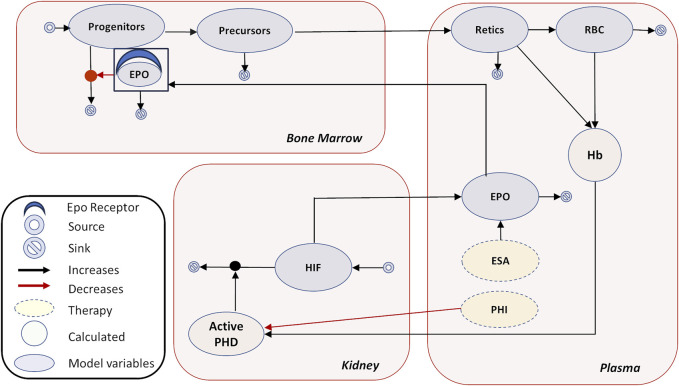
Erythropoiesis model diagram. The model diagram shows the main tissues of interest—plasma, kidney, and bone marrow. The model species are shown in oval—PHIs, HIF, EPO, progenitors, precursors, Retics (reticulocytes), and RBCs. The calculated variables are Hb and active PHD. The major physiological feedback is captured as follows in the model: regulation of PHD by Hb, PHD-mediated HIF degradation, HIF-mediated EPO production, and rescue of erythroid progenitors from apoptosis by EPO. In addition, EPO is distributed to a peripheral compartment, as reported in the PK section, which is not shown here for clarity. No EPO-mediated PD effects take place in the peripheral compartment. Endogenous EPO or externally injected ESAs undergo receptor-mediated degradation upon interaction with EPOR in the bone marrow. Production and degradation of cellular components, as well as continuous proliferation and apoptosis of different cell types, are incorporated. In addition, pharmacokinetics of rHuEPO, darbepoetin, and PHIs are modeled separately with their concentrations matching available PK data in plasma (these PK modules are not shown). Refer to Section 3.1 under Methods for details.

Under healthy physiological conditions, EPO is the major regulator of erythropoiesis and in turn controlled by HIF transcription factors present in the kidneys. HIF consists of the oxygen-sensitive HIF-α subunit and a constitutively expressed HIF-β subunit. Under normoxic conditions, the HIF-α subunits are produced, hydroxylated, and continuously degraded, but under hypoxic conditions, as occurs in anemia, the activities of HIF-PHDs are decreased, degradation of HIF-α is inhibited, and cellular levels of HIF-α are increased. Stabilization of HIF-α allows it to translocate to the nucleus and dimerize with the HIF-β subunit forming the active HIF transcription factor, which leads to the transcriptional activation of target genes involved in erythropoiesis and iron homeostasis (Haase, 2010). HIF-regulated transcription of *EPO* in response to hypoxia leads to an increased production of endogenous EPO by renal interstitial fibroblast-like cells. EPO is rapidly secreted into the plasma and acts on erythropoietin receptors (EPORs) present on committed erythroid progenitor cells in the bone marrow (BM), inhibiting their apoptosis and promoting their differentiation, thereby increasing their descendants which are the Hb-synthesizing erythroblasts, as shown in Figure 1.

In advanced CKD, transformation of interstitial fibroblasts into myofibroblast-like phenotypes renders them incapable of producing EPO (Shih et al., 2018). The HIF-mediated response to chronic hypoxia is diminished in the advanced CKD setting. The kidneys still retain the ability to produce a basal level of EPO, which increases in response to hypoxia (as demonstrated in people living in high-altitude regions) and acute anemia, albeit with a relatively diminished response to the Hb concentration (Brookhart et al., 2008).

PHIs target the HIF-PHD pathway to increase EPO production. Preventing HIF-α prolyl hydroxylation inhibits HIF-α degradation. Thus, PHIs mimic a hypoxic state and increase EPO production. Furthermore, they also improve iron mobilization to the bone marrow (Gupta and Wish, 2017).

### 2.4 Context of the current study among mathematical models of erythropoiesis in the literature

Many mechanistic models that aim to describe the process of erythropoiesis in different mammalian species have been proposed. In brief, previous models focused on the complex dynamics of aging of the precursors, with later models including the pharmcakinetics and pharmacodynamics (PK/PD) of rHuEPO and other ESAs. We have elaborately reviewed some of these approaches in the “Mathematical Models of Erythropoiesis in the Literature” section in the Supplementary Material. More recent models include PHIs and the ability to simulate clinical trials.

Particularly, a QSP model of hematopoiesis was developed on the Entelos PhysioLab platform, which is an ordinary differential equation (ODE)-based mathematical model that captures the key aspects of hematopoietic physiology. Single-dose PK/PD data were used to establish a two-compartment PK model of daprodustat and to represent the response of a PHI to HIF-regulated EPO production (Katy Moore et al., 2011). Recently, InSysBio developed a QSP model that aims to describe the effect of blood donation, chemotherapy, and pharmacological interventions on erythropoiesis for treating anemia. The model describes the various cell processes that hematopoietic stem cells undergo in the bone marrow during maturation into red blood cells (RBCs). The model captures the PK of carboplatin, ESAs, and PHIs and clinical response (serum EPO and reticulocyte count) to the drugs (Alexander Stepanov, 2022). Furthermore, the group compared various methods for virtual population generation in this QSP model: Markov chain Monte Carlo, models fitting to a Monte Carlo sample, population of clones, and stochastically bounded selection (Kolesova et al., 2022). They proposed that among the four approaches, a stochastically bounded selection method yields results with a better representation of experimentally measured mean and standard deviation data and is most applicable in the case of complex models with large numbers of variable parameters.

As observed from the literature survey (as previously mentioned and in the Mathematical Models of Erythropoiesis in the Literature section in the Supplementary Material), comprehensive mechanistic models of erythropoiesis have been proposed already. However, the current model, to the best of our knowledge, is the only open-source QSP model with executable codes and published equations of erythropoiesis with a Vpop that is consistent with several ESA and PHI clinical trials by implementing an adaptive dose titration protocol. It incorporates adequate physiology and mechanistic interactions regulating the process of erythropoiesis in humans such that it can explain the dysregulation of these erythropoietic mechanisms in anemia due to CKD and their response under ESA and PHI applications. This enables us to run simulations of clinical trials of ESAs and PHIs on the model to predict the efficacy of the therapy. To this end, we focused all data and perturbations incorporated in the model to be strictly clinical. Furthermore, we limited the detail in the mechanistic representation to be fit for purpose. For example, although the mechanistic understanding of erythrocyte progression and aging can be critical in understanding the immediate response to an ESA, we focused on capturing chronic (∼weeks), stable response to ESAs, as evidenced by the delays in reticulocyte appearance in the plasma and eventual effect on Hb, and limited the detail in erythrocyte maturation.

The PK model incorporating the drug dose and dosing regimen captures the PK dynamics and drives downstream PD interactions. In this respect, our work is similar to other QSP efforts in the field (Katy Moore et al., 2011; Alexander Stepanov, 2022). To simulate trials, we focused on creating the variability in physiology that reflects in the variability in baseline characteristics (Hb and EPO) and response (measured by Hb time-course) to therapy, as observed in typical phase 2 trials for ESAs (epoetin alfa and darbepoetin alfa) and PHIs (vadadustat and daprodustat). Some model parameters are extracted from the literature, while most of the parameters are estimated by optimizing the model fit to the clinical trial data identified.

Furthermore, adaptive dosing algorithms routinely used for ESAs and PHIs to achieve a target Hb level were implemented, which enables the model to reproduce the clinical trial design faithfully. We also validated the model by predicting the Hb response to different dosing regimens (Q2W and TIW) of rHuEPO (not used to calibrate the model) administered for 44 weeks. The model reliably predicted the Hb profile with different rHuEPO dosing regimens, as well as the average weekly dose needed to achieve the target Hb level with these dosing schedules. The model can be used to visualize and optimize complex clinical trial designs involving adaptive dosing regimens, identifying responders and non-responders, testing mechanistic hypotheses, etc.

## 3 Methods

### 3.1 Modeling approach

The interactions between physiological entities are mathematically represented by ordinary differential equations (ODEs) and implemented in SimBiology 2018a (refer to the Supplementary Material for the model code). The conceptual model focuses on relevant physiological entities which are the plasma, kidney and bone marrow and captures the process of erythropoiesis in adequate mechanistic detail. In addition, the endogenous EPO and exogenous treatment (rHuEPO, darbepoetin, and PHIs) in the plasma also distribute over a generic peripheral compartment that represents other tissues to capture their pharmacokinetics (PK) profile correctly (not shown in Figure 1). Generation of various erythroid precursors through maturation and EPO secretion occurring in the kidney are simplified and not captured in detail as observed in some other mechanistic models of erythropoiesis (Krzyzanski et al., 2005; Schirm et al., 2014) but are fit for purpose to simulate clinical trials.

The model has several alternate parameterizations representing different VPs that have been calibrated to “steady-state” disease of various severities and response to treatment with ESAs and PHIs. Furthermore, based on the description of patient characteristics from clinical trials, we created VPs with CKD not on KRT, i.e., non-dialysis CKD VPs (CKD stages 3 –5), and hemodialysis VPs (only CKD stage 5). The steady state is perturbed by simulating ESA and PHI administration, and the response of the VPs has been calibrated to be consistent with clinical data. Perturbations result in the model reaching a new steady state, with the general time scale of transients ranging from few weeks to months. The fastest time scale is hours, which accounts for variation in EPO levels after ESA administration. Additional considerations and limitations in the model design are discussed in the “Model Design and Equations” section in the Supplementary File.

### 3.2 Design assumptions

a. The model captures disease at a steady state; in other words, for a given stable EPO concentration, the VP will be at a corresponding stable Hb concentration, based on their disease severity. This is a simplification and limits the scope of model prediction to the scale of months (>3 months) given that CKD is a progressive disease where the steady state changes over the course of years. This time scale is comparable to typical phase 2 and 3 trials for CKD anemia and hence fit for that purpose.
b. The model assumes a state of iron sufficiency (anemia due to Fe deficiency is not considered). This is in alignment with our current modeling objective of understanding anemia due to CKD caused by reduced EPO production and shortening RBC lifespan. Dynamics of the EPORs (present on erythroid progenitors) are also not explicitly captured with the assumption that saturation of the receptors does not occur. As often observed in phase 2 and phase 3 trials for CKD anemia, iron sufficiency is a requirement for inclusion in the trial, and the current study was further carried out with that assumption.
c. EPO has known diurnal variability, and this has not been explicitly captured in the model (Cotes and Brozovic, 1982). This is because increases in average EPO over weekly or longer time scales rather than diurnal variability contribute to increasing Hb. This limits the interpretation of simulations of EPO in the time scale of a day or shorter.
d. For patients undergoing hemodialysis, there are likely relevant periodic changes associated with hemodialysis that may impact anemia by alleviating uremia (Bowry and Gatti, 2011). However, in our representation, while the HD-Vpop is more severe, the contribution of blood loss due to hemodialysis to anemia is not captured.

Each of these limitations in design, especially explicit modeling of iron sufficiency, may be addressed in future versions of the model by us or carried out by other interested groups based on the shared model code.

### 3.3 Physiology captured in the model

#### 3.3.1 Hb-PHD-HIF pathway

##### 3.3.1.1 Physiology

Under normoxic conditions, the HIF-α subunit is produced and continuously degraded due to the action of HIF-PHDs. Under hypoxic conditions, the activity of HIF-PHDs is decreased and degradation of HIF-α subunits is inhibited, leading to the transcriptional activation of target genes involved in erythropoiesis and the closely related process of iron delivery to the marrow erythroid cells (McMahon and Singh, 2019).

##### 3.3.1.2 Model representation

The concentration of active PHD is regulated by oxygen saturation, and we modeled in such a way that the concentration of active PHD is directly dependent on the Hb level (Koury and Haase, 2015). This relationship between Hb and PHD is captured using a Hill function such that the active PHD concentration decreases with a decrease in the Hb level (“Model Design and Equations” in Supplementary Material). The presence of PHI further decreases the active PHD concentration. The mathematical equation expressing the dependence of PHD concentration on Hb level and PHI is as follows:

$$PHD=\frac{PHD_{basal}}{\left(1+PHI_{effect}+Hb_{effect}\right)}.$$ (1)

Additional detail about the form of the Hbeffec is given in the “Detailed Equation” section in the Supplementary Material.

The degradation of HIF-α in turn is driven by active PHD concentration, and the half-life of HIF-α varies from 5 to 8 min for normal Hb levels varying from 9 to 14 g/dL for healthy subjects with no external treatments (Berra et al., 2001). In the following equation, *HIF*α and *PHD* represent the dynamic concentration of HIF-α and PHD, respectively, where *PHD* is calculated using the aforementioned Eq. [i].

$$Rate\;of\;HIF\alpha \;\;degradation=\left[HIF\alpha \right]\times \left[PHD\right]\times k_{modulate\_PHD\_effect\_on\_HIF\alpha },$$ (2)

where $k_{modulate\_PHD\_effect\_on\_HIF\alpha }$ is the rate constant that accounts for the effect of active PHD on the HIF-α concentration.

$$\frac{d\left(HIF\alpha \right)}{dt}={kprod}_{HIF\alpha }-\;Rate\;of\;HIF\alpha degradation.$$ (3)

#### 3.3.2 HIF effect on EPO production, its secretion into the plasma, and binding to EPORs

##### 3.3.2.1 Physiology

HIF-regulated transcription of the *Epo* gene in response to hypoxia leads to the production of EPO by renal interstitial fibroblast-like cells. There is a continuous increase in the plasma EPO concentration over a period of 1–2 weeks, which can be attributed to the gradual recruitment of a pool of cells in the kidney for EPO production during hypoxic conditions/phlebotomy (Maeda et al., 1992). This is based on the observation that following phlebotomy, there is no increase in the EPO production per cell, but the number of cells producing EPO increases drastically. The number of EPO-producing cells in the kidney increases exponentially with a reduction in the Hb level and is relatively rapid (hours) (Koury et al., 1989).

##### 3.3.2.2 Model representation

The model consists of the variable *EPO_plasma_* to track the dynamics of EPO in the plasma. The concentration of EPO in the plasma is determined by the rate at which it is produced in the kidney and released into the plasma and its degradation rate. The primary driver of EPO production is HIF-α and is represented by a Hill function dependent on HIF-α concentration. The parameter $k_{production_{EPO}}$ serves as the maximal rate of production.

$$Production\;of\;EPO_{plasma}=\frac{k_{production_{EPO}}\times {HIF\alpha }^{n2}}{{Km_{prod_{EPO}}}^{n2}+{HIF\alpha }^{n2}}.$$ (4)

The removal of EPO from plasma is determined by three factors, namely, non-specific clearance from plasma, net distribution to the peripheral compartment, and target-mediated clearance upon interaction with EPO receptors on the cell species in the bone marrow.

$$Non-specific\;plasma\;clearance=kel_{EPO_{plasma}}\times \left[EPO_{plasma}\right],$$ (5)

$$EPO_{Plasma\_to\_peripheral}=k_{epo_{cp}}\times \left[EPO_{plasma}\right]-k_{epo_{pc}}\times \left[EPO_{periphery}\right],$$ (6)

where $k_{epo_{cp}}$ and $k_{epo_{pc}}$ are EPO distribution rates from plasma to peripheral compartment and *vice versa*, respectively.

The EPO concentration tracked in the plasma is assumed to be the same as the amount reaching the bone marrow (refer to the “Model Design and Equations” section in the Supplementary Material for details). EPORs are expressed on CFU-Es (known as progenitors in the model) that mature into early reticulocytes following EPO–EPOR binding. The saturation of receptors never occurs, and the dynamics is decided by the formation and degradation of the EPO–EPOR complex in the model (similar to the approach considered by Krzyzanski et al., 2005). This is a simplification of the actual physiology of the process. In the bone marrow, EPO binds to free EPORs on erythroid progenitors at the rate $kon_{EPO-LR_{complex}}$ , forming the ligand–receptor complex $EPO-LR_{complex}$ . The complex dissociates at the rate ${koff}_{EPO-LR_{complex}}$ . The binding of EPO to EPORs is a reversible reaction represented by the following rate equation:

$${EPO-EPOR}_{forward\_binding}=kon_{EPO-LR_{complex}}\times \left[EPOR\right]\times \left[EPO_{plasma}\right],$$ (7)

$${EPO-EPOR}_{backward\_binding}=ko{ff}_{EPO-LR_{complex}}\times \left[EPO-LR_{complex}\right],$$ (8)

$$Net\;binding\;of\;EPO\;to\;EPOR=\left(kon_{EPO-LR_{complex}}\times \left[EPOR\right]\times \left[EPO_{plasma}\right]\right)-\left(ko{ff}_{EPO-LR_{complex}}\times \left[EPO-LR_{complex}\right]\right),$$ (9)

$$Total\;Clearance\;of\;EPO_{plasma}=Non\;specific\;plasma\;clearance+EPO_{Plasma\_to\_peripheral}+Net\;binding\;of\;EPO\;to\;EPOR,$$ (10)

$$\frac{d\left({EPO}_{plasma}\right)}{dt}=Production\;of\;EPO_{plasma}-\;Total\;Clearance\;of{EPO}_{plasma}.$$ (11)

The model contains approximately 1,000 EPORs per progenitor cell (CFU-E) (Broudy et al., 1991). Free EPO receptors are modeled in a dynamic way using the following equation, but it is assumed that they never saturate due to constant recycling:

$$\frac{d\left(EPOR\right)}{dt}=\left(kdeg_{EPO-LR}\times \left[EPO-LR_{complex}\right]\right)–\left({EPO-EPOR}_{forward\_binding}\right)+\left({EPO-EPOR}_{backward\_binding}\right).$$ (12)

The interaction between the EPO and EPOR takes place in molecule/volume units, as observed in the following equation. To convert $EPO_{plasma}$ from ng/mL to molecule/mL, the concentration of EPO (in mg/ml) is divided by the molecular weight of EPO, as described in the “Model Design and Equations” section in the Supplementary Material.

$$\begin{array}{ll} \frac{d\left(EPO-LR_{complex}\right)}{dt} & =\left(kon_{EPO-LRcomplex}\times \left[EPOR\right]\times \left[EPO_{plasma\_molecule\_ml}\right]\right)\\ & -\left(ko{ff}_{EPO-LR_{complex}}\times \left[EPO-LR_{complex}\right]\right)-\left(kdeg_{EPO-LR}\times \left[EPO-LR_{complex}\right]\right), \end{array}$$ (13)

where $kdeg_{EPO-LR}$ is the internalization rate of the complex.

#### 3.3.3 Reduced apoptosis of progenitors due to EPO signaling, increased differentiation into reticulocyte precursors, and increased maturation into plasma reticulocytes and RBCs

##### 3.3.3.1 Physiology

The immature lineage-specific progenitor cells are burst-forming unit-erythroids (BFU-Es), followed by CFU-Es present in the bone marrow. CFU-Es possess EPORs, and EPO binding to EPORs protects the cells from apoptosis, thereby promoting its differentiation to pro-erythroblasts. Pro-erythroblasts further undergo differentiation into bone marrow reticulocytes, after which they enter the circulation (Elliott et al., 2008). It takes 1–2 days for blood reticulocytes to mature into erythrocytes. The entire process of maturation of human EPO-responsive progenitors to RBCs typically takes approximately 8–10 days (Ramakrishnan et al., 2004).

##### 3.3.3.2 Model representation

The model consists of progenitor species in the bone marrow represented (named progenitors in the model) that mature into premature reticulocytes (named precursors in the model) that are eventually released into the plasma, forming plasma reticulocytes (named retics in the model). The effect of EPO signaling on progenitors is modeled by a Hill function, which decreases the rate of apoptosis of progenitors. The model simplifies the multiple stages of erythrocyte maturation with only four variables which represent the different stages of maturation that a progenitor cell undergoes. Although this is adequate to match the dynamics of reticulocytes, RBCs, and Hb level in response to the perturbations of interest, it is not designed to capture the specific short-term dynamics of precursor maturation.

$$Production_{progenitors}=k_{prod\_Progenitors},$$ (14)

$$Maturation_{progenitors}=k_{ProgenitorsToPrecursors}\times \left[Progenitors\right].$$ (15)

The rate of degradation of progenitors is downregulated with the EPO–EPOR interaction in their surface (“Model Design and Equations” section in the Supplementary Material).

$$Degradation_{progenitors\_regulated\_by\_EPO-EPOR}=k_{baseline\_deg}\times \left[Progenitors\right]\times \left(1-\;f\left(EPO-LR_{complex}\right)\right),$$ (16)

$$\frac{d\left(Progenitors\right)}{dt}=Production_{progenitors}-Maturation_{progenitors}-Degradation_{progenitors\_regulated\_by\_EPO-EPOR}.$$ (17)

In the model, the variable “precursors” is used as a surrogate for bone marrow reticulocytes.

$$Production_{precursors}=k_{progenitorsToPrecursors}\times \left[Progenitors\right],$$ (18)

$${Degradation}_{precursors}=k_{\deg\_precursors}\times \left[Precursors\right],$$ (19)

$$Release\;of\;Precursors\;to\;plasma=k_{PrecursorsToRetics}\times \left[Precursors\right],$$ (20)

$$\frac{d\left(Precursors\right)}{dt}=Production_{precursors}-{Degradation}_{precursors}-Release\;of\;Precursors\;to\;plasma.$$ (21)

A delay, representative of the maturation period, is introduced between precursors (bone marrow reticulocytes) and the appearance of reticulocytes in circulation based on the literature data on the appearance of reticulocytes with ESA administration (Jelkmann, 2004). Specifically, the maturation rate is determined by calibrating to observations from clinical trials; for example, the trial involving administration of a high dose of 40,000 IU QW rHuEPO to healthy subjects that results in an increase in the number of plasma reticulocytes over a week, peaking on day 10, long after the rHuEPO peak is observed (Tmax of 16 h after exogenous EPO dose) (Cheung et al., 2001).

$$Maturation_{precursors\_to\_retics}=k_{PrecursorsToRetics}\times \left[Precursors\right],$$ (22)

$$Maturation_{retics\_to\_RBCM}=k_{ReticsToRBCM}\times [Retics_{\left.plasma\right]},$$ (23)

$${Degradation}_{Retics_{plasma}}=k_{\deg\_Retics_{plasma}}\times \left[Retics_{plasma}\right],$$ (24)

$$\frac{d\left(Retics_{plasma}\right)}{dt}=Maturation_{precursors\_to\_retics}-Maturation_{retics_{\_to_{\_RBCM}}}-{Degradation}_{Retics_{plasma}},$$ (25)

$${Degradation}_{RBCM}=k_{\deg\_RBCM}\times \left[RBCM\right],$$ (26)

which results in

$$\frac{d\left(RBCM\right)}{dt}=Maturation_{retics\_to\_RBCM}-{Degradation}_{RBCM}.$$ (27)

#### 3.3.4 Calculating the concentration of Hb from RBC and reticulocyte count

##### 3.3.4.1 Physiology

MCH_Reti and MCH_RBCM are constants that represent the concentration of Hb per reticulocyte and RBC, respectively (Brugnara, 2000; Mast et al., 2002). The mean corpuscular hemoglobin in reticulocytes and RBCs may vary across CKD stages due to the prevalence of nutritional deficiencies like iron, vitamin B12, and folic acid, or other causes of hemolysis and bleeding (microcytosis vs. macrocytosis vs. normocytosis) (Mikhail et al., 2017). However, as the model assumes iron sufficiency, these are currently not varied across CKD stages. The net Hb is a function of both the number of these cells (reticulocytes and RBCs) and the concentration of Hb they carry.

##### 3.3.4.2 Model representation

The Hb concentration changes in the model as a function of the concentration of reticulocytes and RBCs.

$$HGB=MCH\_Reti\times \left[Retics_{Plasma}\right]+MCH\_RBCM\times \left[RBCM\right].$$ (28)

Finally, this in turn regulates the PHD, completing the feedback loop (Figure 1).

### 3.3.5 Drug Pharmacokinetics (PK)

rHuEPO and darbepoetin alfa are large glycoprotein biologic ESAs which can be administered intravenously or subcutaneously. A two-compartment model consisting of the plasma (central) compartment and peripheral compartment is used to model drug PK. Clearance of the intravenously administered ESA is represented by a plasma elimination rate constant, $kel_{EPO\_plasma}$ , and through the incorporation of parameters pertaining to target-mediated drug disposition (TMDD). Implementation of the TMDD component is essential as it explains the nonlinear PK observed with ESAs. Both rHuEPO and darbepoetin bind to EPORs on erythroid progenitors to form the EPO–EPOR complex. This complex is internalized by the EPOR-expressing progenitors, which is modeled using an internalization rate parameter ( $kdeg_{EPO-LR}$ ). $kdeg_{EPO-LR}$ is the same for both the ESAs.

Subcutaneously administered ESA is implemented using a first-order absorption rate constant (*ka*) depicting absorption into the plasma. The rate of absorption of the drug into the plasma is lower than the rate of elimination, which makes *ka* the rate-limiting parameter. After absorption, the drug is distributed to the peripheral tissues from where it again recirculates back to the plasma. This distribution is taken into account by the parameters $k_{epo_{cp}}$ and $k_{epo_{pc}}$ . While this is a simplification of complex pharmacokinetic behavior (Ramakrishnan et al., 2004; Woo et al., 2007), this model structure is adequate to capture the drug effect on the physiology over a chronic time period where exposure limitation is not considered to be a major factor in the limitation of efficacy.

The PK data showing the time profile of drug concentration after the administration of rHuEPO and darbepoetin alfa were obtained from Cheung et al. (2001) and Kim et al. (2019), respectively, and were used to calibrate the model (Cheung et al., 2001; Kim et al., 2019).

The PK profile of PHIs are captured by a simple two-compartment model with delayed first-order absorption into the plasma and first-order clearance. The parameter CL is used to represent tissue distribution and recirculation. They are modeled using parameters that account for its absorption into plasma and elimination from the central compartment, as demonstrated in Singh et al. (2015). However, the biphasic behavior observed in the PK of daprodustat is captured using an additional nonlinear clearance from the plasma. The information on plasma PK for vadadustat and daprodustat was obtained from Chavan et al. (2021) and Yamada et al. (2020), respectively. Additional information on the PK data is given in Table 1.

**TABLE 1:** List of articles selected for model calibration to the PK of ESAs and PHIs. Details about drug, dose, and trial population are reported.

Paper	Population type	Drug (route of administration)	Dose
PK calibration			
Cheung et al. (2001)	Healthy volunteers	rHuEPO (SC)	40,000 IU QW and 150 IU/kg TIW
Kim et al. (2019)	Healthy volunteers	Darbepoetin alfa (SC)	60 μg (single dose)
Chavan et al. (2021)	Healthy volunteers	Vadadustat (QD)	450 mg (single dose)
Yamada et al. (2020)	Healthy Japanese male volunteers	Daprodustat (QD)	4 mg (single dose)

**FIGURE 2:**
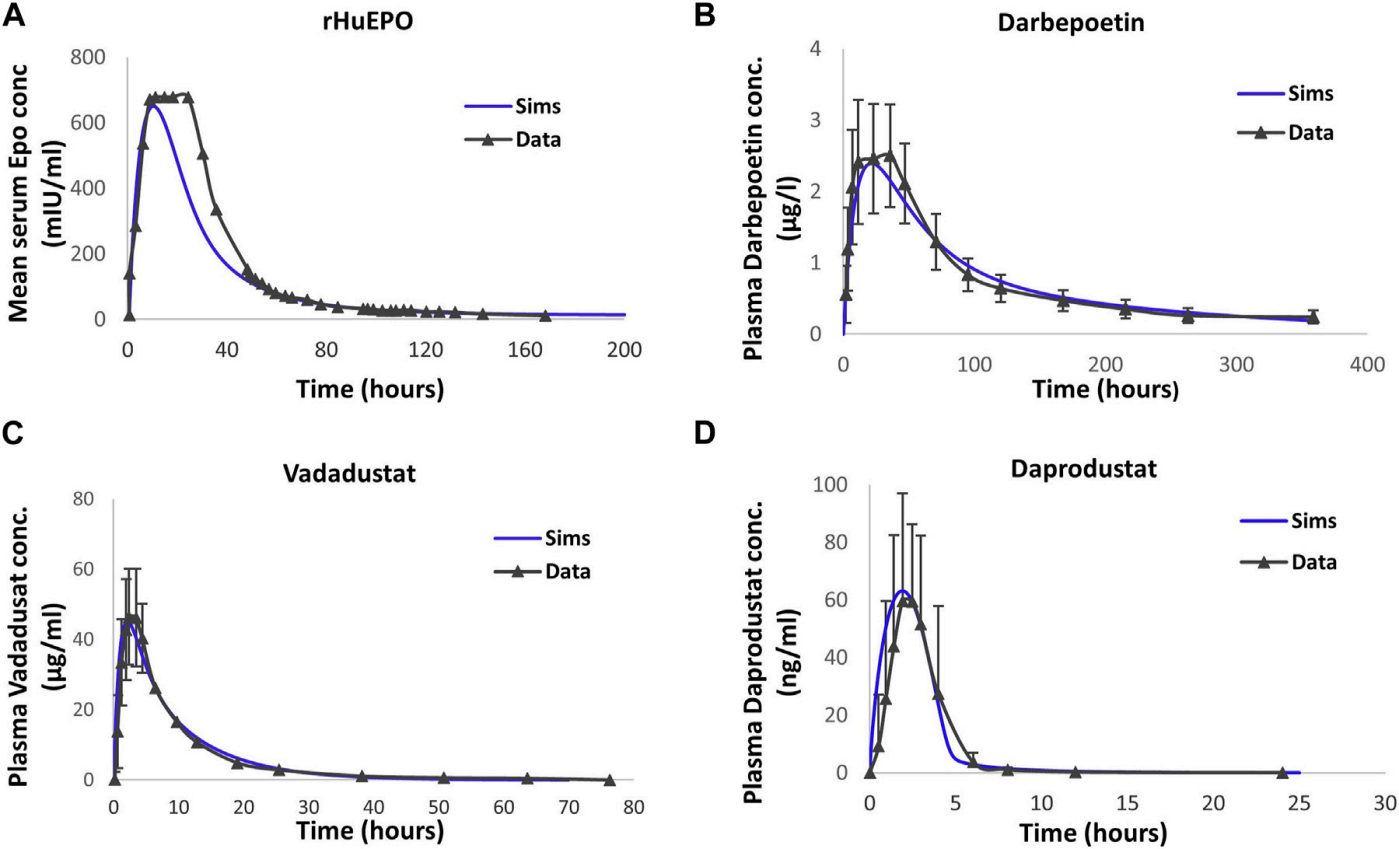
Model calibration to the pharmacokinetic time profile data of therapies. Detailed information on subjects, dose, and observation time points are provided in the “Drug Pharmacokinetics” subsection in Methods. The blue solid line corresponds to model simulations. rHuEPO **(A)**, darbepoetin **(B)**, vadadustat **(C)**, and daprodustat **(D)** data are represented by black solid lines with markers. Data are represented as the mean ± standard deviation. Note that SD for plasma EPO concentration with rHuEPO is not reported in the paper.

Figure 2 shows the simulation results after model calibration to the PK of ESAs and PHIs. The PK parameters are tabulated in the “Parameter Dashboard” section in the Supplementary Material.

### 3.3.6 Drug Pharmacodynamics (PD)

In the event of ESAs being administered to a patient, the plasma EPO is a combination of the endogenous EPO produced by the kidney and ESA (rHuEPO or darbepoetin). The binding properties of rHuEPO are assumed to be the same as those of endogenous EPO, which is reflected using parameters such as *kon*, *koff*, and *kel*. Darbepoetin, on the other hand, has a reduced binding affinity to EPOR and higher *in vivo* activity (Egrie et al., 2003). To account for this difference in receptor binding affinity, *kon* and *koff* values are different for darbepoetin. Both ESAs bind to EPORs, which leads to the formation of the EPO–EPOR complex that helps in the survival of a greater number of erythroid progenitors. In the model, the degradation rate of erythroid progenitors is a function of the EPO–EPOR complex.

The pharmacodynamic effect of PHI administration is modeled as reducing the activity of PHD, as shown in Eq. (i). Thus, PHIs exert their therapeutic activity by modulating the HIF-PHD pathway upstream of EPO.

### 3.4 Model calibration

#### 3.4.1 Model parameters

Model parameters determine the strength and form of the interaction between various species of the model and are fixed for every VP generated in the model. Deriving parameter values from *in-vitro* and pre-clinical *in-vivo* experiments that were conducted on animals like mice were deemed unsuitable since the values are not likely to be the same across species. Therefore, the model consists of only human parameters like RBC lifespan and reticulocyte maturation time, which have been obtained from the literature. In the absence of reliable literature sources, a single set of parameter values has been estimated to fit clinical outcome data from multiple trials and optimized during model calibration. The model parameters are summarized in the Supplementary Material (Parameter Dashboard), where the details of the parameters those directly used from data and those estimated to reach the appropriate “top-down” clinical trial behavior are shown.

#### 3.4.2 Creating VPs of varying disease severities

A reference VP is a parameterization of the physiology in the model such that they reproduce mean clinical readouts of the disease at the steady state and in response to therapy. Five distinct VPs are created—one healthy and four patients—with increasing severity of both CKD and anemia. CKD progression is associated with reduced HIF-mediated production of EPO, reduced production and increased degradation rate of progenitors, and increased degradation rate of mature RBCs (Li et al., 2019). Therefore, the parameters representing the aforementioned physiology are varied in order to create reference VPs of varying severities. Supplementary Table S1 shows the parameters varied to create the VPs for each CKD stage.

Although two distinct classifications exist for CKD 1 and CKD 2, we created CKD 1.5, which is an average representation of the two disease stages, as anemia in CKD stage 1 or 2 is usually extra-renal in etiology. Furthermore, most of the clinical trials targeting anemia due to CKD recruit patients with CKD 3, CKD 4, and CKD 5, and data were inadequate to delineate CKD 1 and CKD 2. Therefore, CKD 1.5 reference VPs are only generated for constraining physiological parameters and defining the continuity (progression from CKD 1 to CKD 5), but it is not part of any Vpop. Clinical data show that the Hb level decreases steadily as CKD progresses, with CKD Stage 5 patients having an Hb level less than 10 g/dL. The plasma erythropoietin level, on the other hand, is not a reliable clinical readout for quantification of anemia due to CKD. This is because CKD 3 or CKD 4 patients may have plasma EPO levels equivalent to those observed in healthy volunteers.

#### 3.4.3 Simulating steady-state dynamics and response to therapy in VPs

The physiological parameters of the healthy VP and four CKD VPs are identified to align with reported values under baseline (unperturbed) conditions as well as under perturbed conditions with ESA and PHI administration. The data used to calibrate the VPs are plasma Hb and plasma EPO levels in healthy and CKD patients (CKD Stages 1–5) with anemia, as reported in the literature.

**FIGURE 3:**
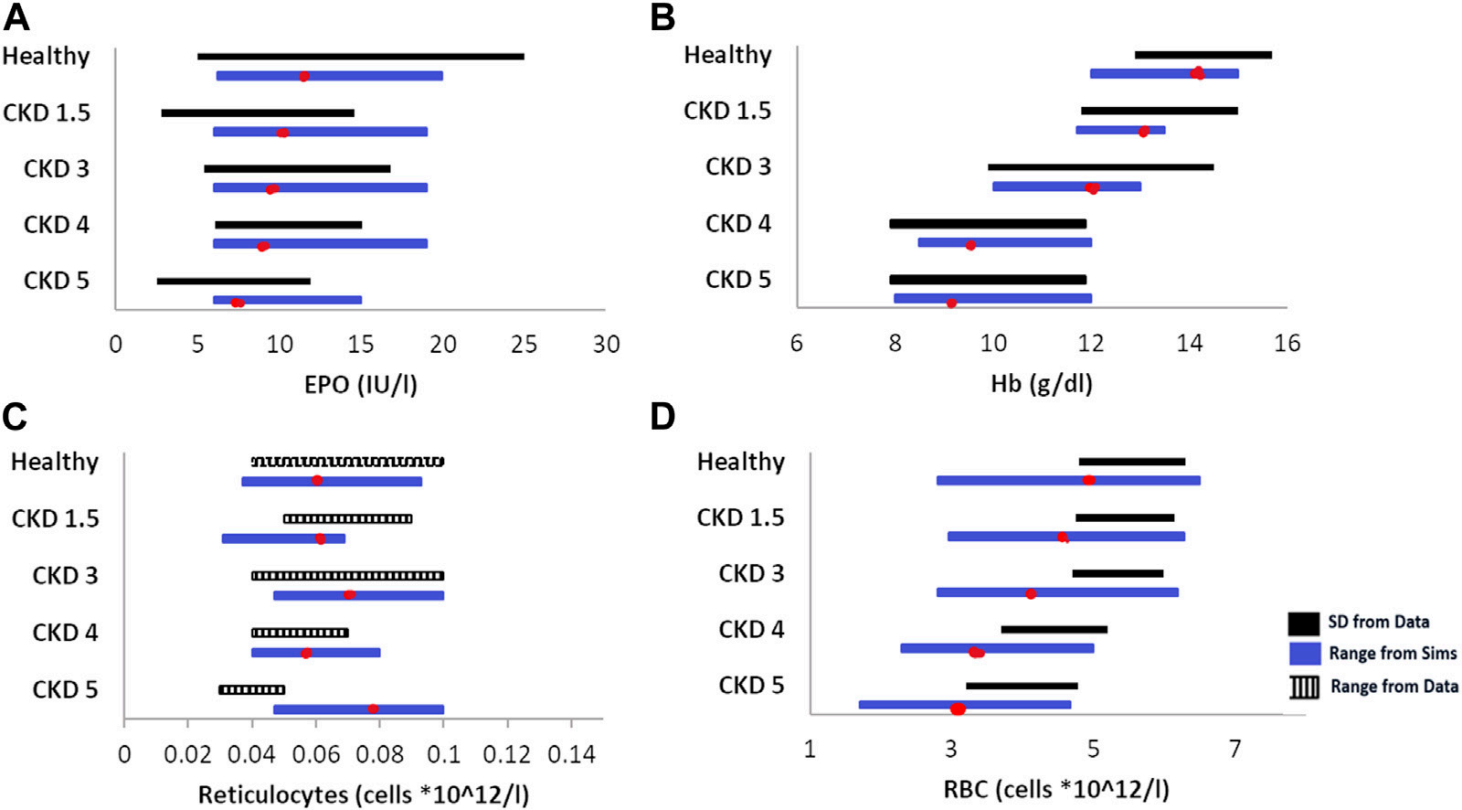
Data vs. simulation comparison of steady-state, unperturbed (without any therapy) values of EPO **(A)**, Hb **(B)**, reticulocytes **(C)**, and RBCs **(D)** in various healthy and anemic patients of varying CKD stages. The solid black bars represent data expressed as the mean ± standard deviation, the striped, black bars represent data expressed as range, and the blue solid bars are simulations (range). The red dot on the simulation bar represents the values of model outputs in different reference VPs in the model. The EPO, Hb, and retic data are obtained from Li et al. (2019); the RBC data are obtained from Sheth et al. (2016).

The diseased anemic steady states are of varying severities and caused by varying underlying pathophysiological factors, as explained in the previous section. Figure 3 shows the range of unperturbed data of the readouts of interest in healthy and CKD patients. This reported range is compared to the reference VP (shown as a red dot), which is calibrated to match the mean value reported for each disease stage.

Furthermore, VPs are also subjected to the following perturbations and calibrated to match the published data of the following therapeutics:

• ESAs (rHuEPO and darbepoetin) on healthy and CKD patients (Nissenson et al., 2002; Locatelli et al., 2003; Suranyi et al., 2003; Provenzano et al., 2004).
• PHIs (vadadustat and daprodustat) on CKD patients (Pergola et al., 2016; Haase et al., 2019; Holdstock et al., 2019; Meadowcroft et al., 2019).

**FIGURE 4:**
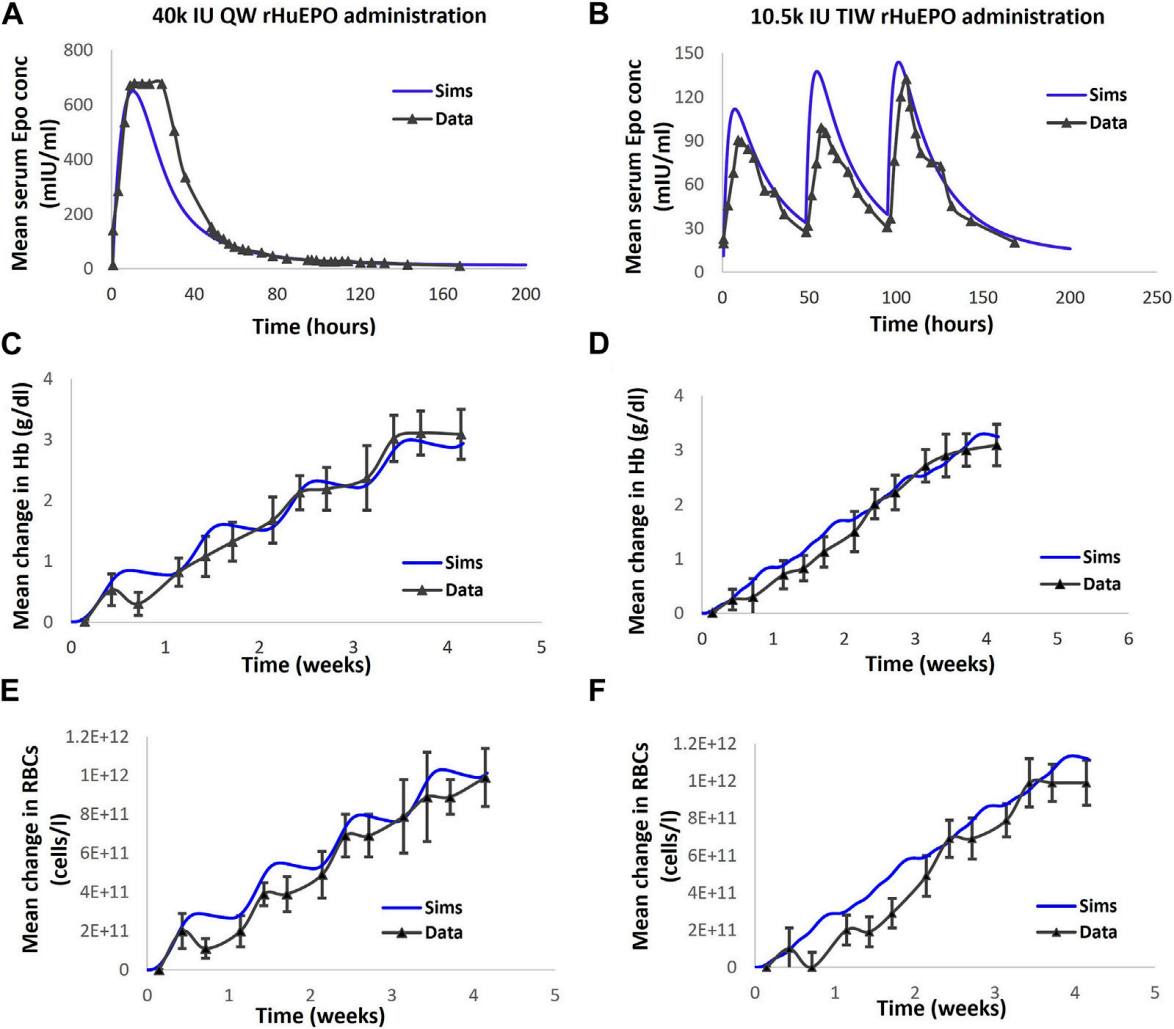
The model is calibrated to clinical readout data of EPO, Hb and RBC in healthy volunteers subjected to rHuEPO administration. **(A, C, E)** correspond to EPO, Hb and RBC dynamics respectively following 40000 IU QW rHuEPO administration. **(B, D, F)** correspond to Epo, Hb and RBC dynamics respectively following the 10.5 k IU TIW dose. Blue solid line represents model output; black solid line with markers is the corresponding data, with mean + standard deviation. Note: Cheung et al., 2001 also reports reticulocytes dynamics with rHuEPO administration. However, the model calibration did not include Reticulocytes, consequently, the comparison of data and simulations for Reticulocytes is omitted.

In particular, the response of the healthy VP to 40,000 IU QW and 10,500 IU TIW in terms of EPO and changes in RBC and Hb is shown in Figure 4. The response of the CKD reference VPs to therapies of interest is shown in the “Developing Virtual Patients” section in the Supplementary Material (Supplementary Figures S2, S3).

#### 3.4.4 Developing virtual populations—ND and HD

A key goal of this effort is to enable visualization of clinical trials with varying baseline characteristics and trial designs. To accomplish that, Vpops (ensembles of VPs) are created to simulate published ESA and PHI clinical trials.

While creating a Vpop for anemia trials in CKD patients, the classification of patients is based on the dialysis status. Patients are either non-dialysis (ND) (either CKD Stages 3, 4, or 5) or more severe hemodialysis (HD) patients (only CKD Stage 5). Based on the described inclusion criteria in the selected trials, we assumed that the ND VP has no prior ESA experience. HD VPs, on the other hand, are on individualized doses of ESAs to stabilize the Hb level (i.e., the baseline Hb of an HD VP is reached due to continued ESA administration) and are yet anemic. It is worth noting that no additional parameters (apart from the parameters in Supplementary Table S3, as stated previously) were varied to differentiate between ND and HD Vpops.

Several approaches exist to create Vpops in QSP workflows (Allen et al., 2016; Cheng et al., 2017; Hosseini et al., 2020), and ours is similar to other approaches (Klinke, 2008; Yu et al., 2021) in the literature, which generates variability around a reference VP to create a Vpop that matches the baseline conditions of the trial (Supplementary Figure S4). More details about the steps taken to create these Vpops are provided in the “Developing Virtual Population” section in the Supplementary Material.

The entire list of trials used to create the Vpops of interest is provided in Table 2, which particularly shows the baseline characteristics of the patients in a trial and their response to specific therapies at the end of the trial. The Vpops for ND and HD are generated to capture the trials.

**TABLE 2:** List of clinical trials selected for the calibration of ND and HD virtual populations in the model. Details about therapy, patient characteristics, baseline conditions, and response to therapies (ESAs and PHIs) of the patients are reported.

Paper	Population type (% of different CKD stages)	Dialysis status (ND or HD)	Drug (route of administration)	Starting dose	Baseline Hb (g/dL) (mean ± SD)	Hb after therapy (g/dL) (mean ± SD)
Calibration						
Cheung et al. (2001)	Healthy	NA	rHuEPO (subcutaneous)	40,000 IU QW; 10,500 IU TIW	13.5 + 0.79	16.4 ± 0.41
Provenzano et al. (2004)	CKD 3: 9.8%; CKD 4: 60.3%; CKD 5: 29.9%	Non-dialysis and treatment-naive	rHuEPO (subcutaneous)	10,000 IU QW	9.1 + 0.7	11.8 ± 1.3
Nissenson et al. (2002)	CKD (stages not reported)	Hemodialysis and treated with rHuEPO	rHuEPO (intravenous)	rHuEPO dose determined by the previous ESA dose	11.23 + 1.43	11.27 ± 2.34
Suranyi et al. (2003)	CKD 3, 4, and 5 (proportions not mentioned)	Non-dialysis and treatment-naive	Darbepoetin (subcutaneous)	10, 15, 20, 30, 40, 50, 60, 80, and 100 µg Q2W	9.9 + 0.9	12.0 ± 1.3
Locatelli et al. (2003)	CKD 5: 100%	Hemodialysis and previously treated with ESAs	Darbepoetin (subcutaneous)	Initial dose based on the rHuEpo dose at the time of entry into the study	11.2 + 0.8	11.3 ± 1.03
Pergola et al. (2016)	CKD 3: 26.1%; CKD 4: 61.6%; CKD 5: 12.3%	Non-dialysis and treatment-naive	Vadadustat (oral)	450 mg QD	9.9 + 0.86	10.8. SD not available
Haase et al. (2019)	CKD 5: 100%	Hemodialysis and previously treated with ESAs	Vadadustat (oral)	300 mg QD	10.4 + 0.7	10.3 ± 0.97
Holdstock et al. (2019)	CKD 3: 22%; CKD 4: 47%; CKD 5: 31%	Non-dialysis and treatment-naive	Daprodustat (oral)	1, 2, or 4 mg QD	10.15 + 0.96	10.98 ± 1.34
Meadowcroft et al. (2019)	CKD (stages not reported)	Hemodialysis and previously treated with ESAs	Daprodustat (oral)	4, 6, 8, 10, and 12 mg QD	10.28 + 0.12	10.38 ± 1.2
Validation						
Pergola et al. (2009)	CKD 3 and CKD 4 (proportion not mentioned)	Non-dialysis and treatment naive	rHuEPO (QW, Q2W, and TIW)	TIW: 50 IU/kg; QW: 10,000 IU; Q2W: 20,000 IU	TIW: 9.63 + 0.86; QW: 9.71 + 0.74; Q2W: 9.91 + 0.75	TIW: 11.38 + 1.04; QW: 11.12 + 1.03; Q2W: 11.19 + 0.97


Each ND Vpop created is matched to have the same fraction of CKD 3, CKD 4, and CKD 5, as reported in the patient inclusion details in the trial (for example, the vadadustat clinical trial had 26.1% CKD Stage 3 patients, 61.6% CKD Stage 4 patients, and 12.3% CKD Stage 5 patients). Each HD Vpop, on the other hand, as reported in the data, consists of only CKD 5 patients on KRT/dialysis (ESRD).

To account for prior administration of ESAs, an additional step is used to generate HD Vpop. This is carried out by choosing a CKD 5 VP and then subjecting it to a range of rHuEPO doses and dosing schedules (3,500–4,800 IU TIW or 4,650 IU QW) to achieve the baseline Hb range reported for the trial.

Furthermore, an ND Vpop is also validated using rHuEPO trial data, which was not used by the model for training purposes (Pergola et al., 2009). The specified trial design and entry criteria for baseline Hb for the ND patients are implemented, but no other changes are made to the model physiology or patient parameters.

#### 3.4.5 Modeling adaptive clinical trials

The trials of therapies targeting anemia due to CKD are all adaptive in nature. This is to maintain the Hb level within the safe target band as an increase in the Hb level (usually Hb > 13 g/dl) is unsafe for the patient (Rosner and Bolton, 2008). The starting dose is decided and then administered to all the trial participants for 2–4 weeks. This is the fixed dose phase of the trial, where dose titration is usually not allowed. The Hb response of each individual patient is closely monitored during this phase. At the end of this phase, the dose that each participant will be receiving is determined by the participant’s specific Hb response to therapy. Generally, the dose is increased if the Hb does not increase by 1 g/dL in 4 weeks. On the other hand, the dose is decreased if a sharp increase in Hb concentration is observed, i.e., if Hb increases by > 1.2 g/dL in 2 weeks. Treatment is withheld, and the patient is closely monitored if Hb exceeds 13 g/dL. The protocol details have been simulated in a similar manner as possible to the description in each publication to reproduce the clinical trial.

However, in the case of the vadadustat ND trial, it is observed that the model fit to the Hb data is better if a slightly modified protocol is employed (Pergola et al., 2016).

The details of the protocol reported are as follows: the starting dose is 450 mg QD (other available doses are 150, 300, and 600 mg QD). The Hb level is monitored over 2 weeks and then adjusted in the following manner based on the response:

If the Hb level reaches 11–12 g/dL and ΔHb > 1 g/dL in 2 weeks, then the dose is switched to the next lower dose.

If the Hb level reaches 11–12 g/dL and ΔHb < 1 g/dL in 2 weeks, then the dose is switched to the next higher dose.

If Hb > 13 g/dL, the dose is withheld.

Thus, the protocol in the clinical trial dictates withholding the dose when the Hb level reaches 13 g/dL. However, simulations suggest that the simulated Vpop is a better match to the data if the vadadustat dose is withheld when the Hb level surpasses 12 g/dL rather than 13 g/dL, as shown in Figure 8.

Next, we generated vadadustat dose–Hb response plots for two HD VPs that are based on the current guidelines that mandate a lower target Hb level of 10–11 g/dL in the US. The dose titration protocol employed is the same as the protocol reported for the vadadustat phase 3 clinical trial in the HD population (Eckardt et al., 2021). The details of the protocol reported are as follows: the starting dose is 300 mg QD with doses of 150, 450, and 600 mg available for adjustment. The current Hb level of each patient determines the adjustment needed to achieve the target Hb level, which is specified as 10–11 g/dL for the US population.

First, the current Hb level is measured to check whether it is < 10 g/dL, 10–11 g/dL, or >11 g/dL. Then, based on whether there is a rapid increase in the Hb level (defined by > 1 g/dL in a 2-week period or >2 g/dL increase in a 4-week period), the dose is adjusted. An Hb level of <10 g/dL and an absence of a rapid increase in the Hb level require an increase in dose by one tablet. An Hb level of 10–11 g/dL and an absence of the rapid increase in the Hb level require dose maintenance. However, if Hb < 11 g/dL and there is a rapid increase in the Hb level, the dose is reduced by one tablet or maintained. The dose is withheld if Hb > 11 g/dL and is resumed with one fewer tablet only after the Hb levels decreases to below 11 g/dL. More details about the vadadustat dose adjustment protocol for the phase 3 HD clinical trial are provided in Supplementary Figure S5.

In clinical trial publications, often, specific information on the trial protocol (for example, washout period and previous rHuEPO dosing) is not reported explicitly. When these protocols are strictly enforced in the model, it may not simulate the physician’s decisions that may have led to variations in the written protocol. For example, Locatelli et al. (2003) decided the initial dose of darbepoetin alfa based on the rHuEPO dose at the time of entry into the study, using a formula equating the peptide mass of the two molecules (200 IU rHuEPO = 1 µg darbepoetin alfa) (Locatelli et al., 2003). The specific rHuEPO dose received by the HD patient on established dialysis is therefore unknown to us. In this scenario, we implemented a strategy wherein we estimated the stable rHuEPO dose at the time of entry into the clinical trial. This is carried out by administering an rHuEPO dose for 4–5 months (dosing frequency similar to that reported in the paper) such that the baseline Hb level (reported at the start of darbepoetin administration) is achieved. Lack of compliance and iron insufficiency play a significant role in determining the Hb profile upon drug administration, which needs to be accounted for while evaluating the model predictions.

## 4 Results

We developed a QSP model capturing the dynamics of erythropoiesis with and without therapy administration in humans. The model map, as shown in Figure 1, captures the physiological mechanism of the production of RBCs and Hb as well as the Hb–PHD–HIF–EPO-regulated feedback loop. (Supplementary Figure S2 in the “Model Design and Equations” section in the Supplementary Material depicts the general quantitative relationships assumed between the key entities of the model).

The model parameters are varied to create a cohort of virtual patients of varying disease severities. The parameters varied to create these patients, and the ranges across which they were varied are given in Supplementary Table S1. A combination of factors, such as reduced EPO production ability and increasing degradation rate of RBCs, contributes to a significant reduction in Hb and RBCs associated with progressive CKD. Figure 2 shows the comparison of the steady-state ranges of plasma EPO, Hb, reticulocytes, and RBCs across healthy VPs and CKD 1–5 as observed in simulations against patient data. Even though the model parameters were not tuned to match the reticulocyte and RBC ranges for individual stages of CKD, the steady-state values from the model are close to the data obtained from Li et al. (2019) and Sheth and Shah (2016), except reticulocytes for CKD 5 (Nidhi Sheth, 2016; Li et al., 2019). This discrepancy in reticulocyte counts among CKD Stage 5 patients can be attributed to the variability in reticulocyte distribution in CKD patients. Better fits to the reticulocyte count may be achieved in future refinements of the virtual cohort by increasing the degradation rate of reticulocytes in the bone marrow and circulation and correspondingly decreasing the degradation of RBCs for CKD 5 patients.

The PK data of ESAs and PHIs of interest are obtained from publicly available data (Table 1), and a two-compartment PK model is used to fit the plasma drug exposure. PK parameters were estimated during model calibration to the PK data and are subsequently kept constant in order to conduct data fitting for the pharmacological responses. As shown in Figure 4 and Supplementary Material (Parameter Dashboard), the rHuEPO PK data are subjected to simultaneous fitting for both 40,000 IU QW and 10,500 IU TIW to determine the PK parameters. For all the drugs, more emphasis has been placed on capturing the terminal phase of the PK as it has the most impact on dosing frequency and efficacy.

**FIGURE 5:**
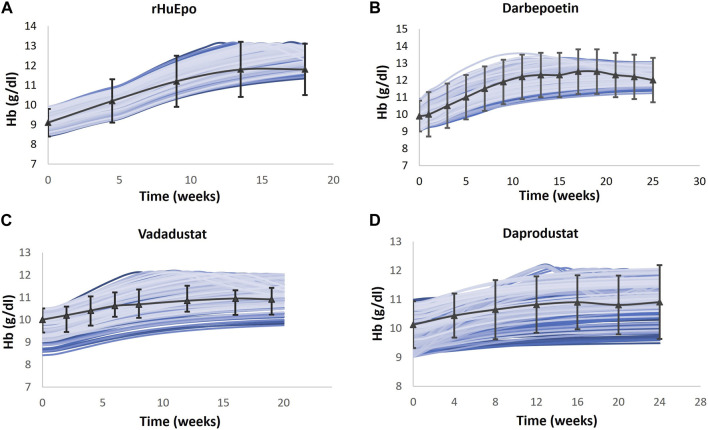
Comparison of models and data for Hb response following therapy in the ND Vpop. Four different ND Vpops representing the population in rHuEPO **(A)**, darbepoetin **(B)**, vadadustat **(C)**, and daprodustat **(D)** are created, and the Hb response to the four therapies is simulated. Blue solid lines indicate the Vpop simulations, wherein each blue line represents a single virtual patient’s response to the drug. rHuEPO, darbepoetin, and daprodustat data are represented by black solid lines with markers, which are expressed as the mean ± standard deviation. Vadadustat data are represented by a black solid line with error bars, which corresponds to the median, 25th percentile, and 75th percentile (Suranyi et al., 2003; Provenzano et al., 2004; Pergola et al., 2016; Holdstock et al., 2019).

**FIGURE 6:**
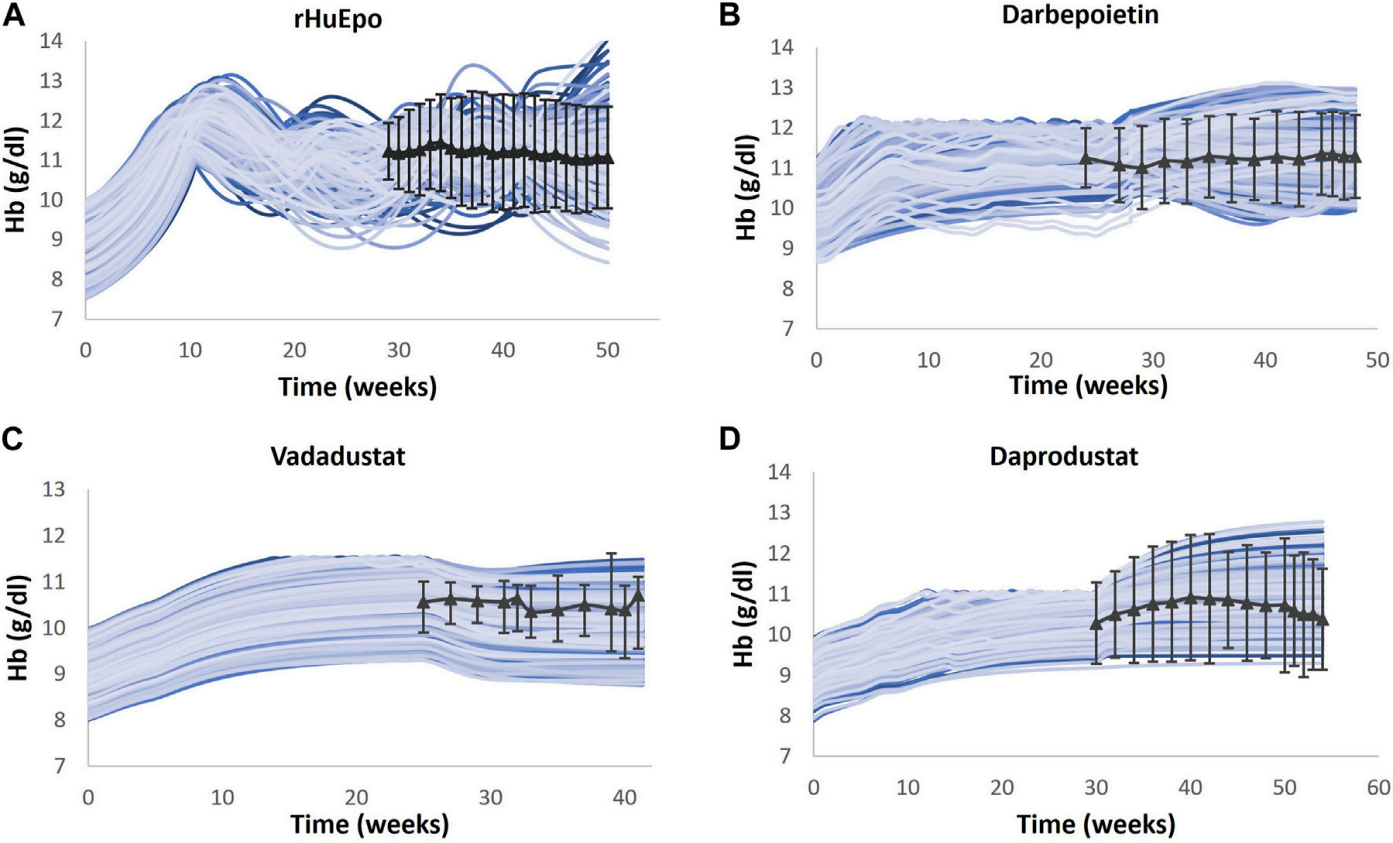
Comparison of models and data for Hb response following therapy in the HD Vpop. Four different HD Vpops representing the population in rHuEPO **(A)**, darbepoetin **(B)**, vadadustat **(C)**, and daprodustat **(D)** are created, and the Hb response to the four therapies is simulated. Blue solid lines indicate the Vpop simulations, wherein each blue line represents a single virtual patient’s response to the drug. rHuEPO, darbepoetin, and daprodustat data are represented by black solid lines with markers, which are expressed as the mean ± standard deviation. Vadadustat data are represented by a black solid line with error bars, which corresponds to the median, 25th percentile, and 75th percentile (Nissenson et al., 2002; Locatelli et al., 2003; Haase et al., 2019; Meadowcroft et al., 2019).

Multiple clinical trials of ESAs are simulated (Table 2), and for each trial, a Vpop that matches the exact baseline characteristics is selected (for more details, refer to the “Developing Virtual Population” section in the Supplementary Material). Ideally, a single Vpop that matches all trials is used in QSP platform approaches (Tanvi Joshi et al., 2021; Dinesh Bedathuru et al., 2023). However, in this case, we chose to create a Vpop for each of these studies to be consistent with the varying entry criteria in each of these studies, which influences the response to the ESA or PHI. In future uses of this model, either a pre-existing Vpop used for one of the trials may be used for predictive simulations (for example, an ND Vpop similar to that recruited for darbepoetin (Suranyi et al., 2003) or a Vpop for a new trial may be created by specifying the entry criteria [BL Hb level and use of ESA]) in the script. As shown in Figure 5, the Hb responses of ND Vpops to rHuEPO, darbepoetin, vadadustat, and daprodustat are simulated, and they are a good match to trial data. Furthermore, as shown in Figure 6, the Hb response of HD Vpops to rHuEPO, darbepoetin, daprodustat, and vadadustat fits the trial data reasonably well.

**FIGURE 7:**
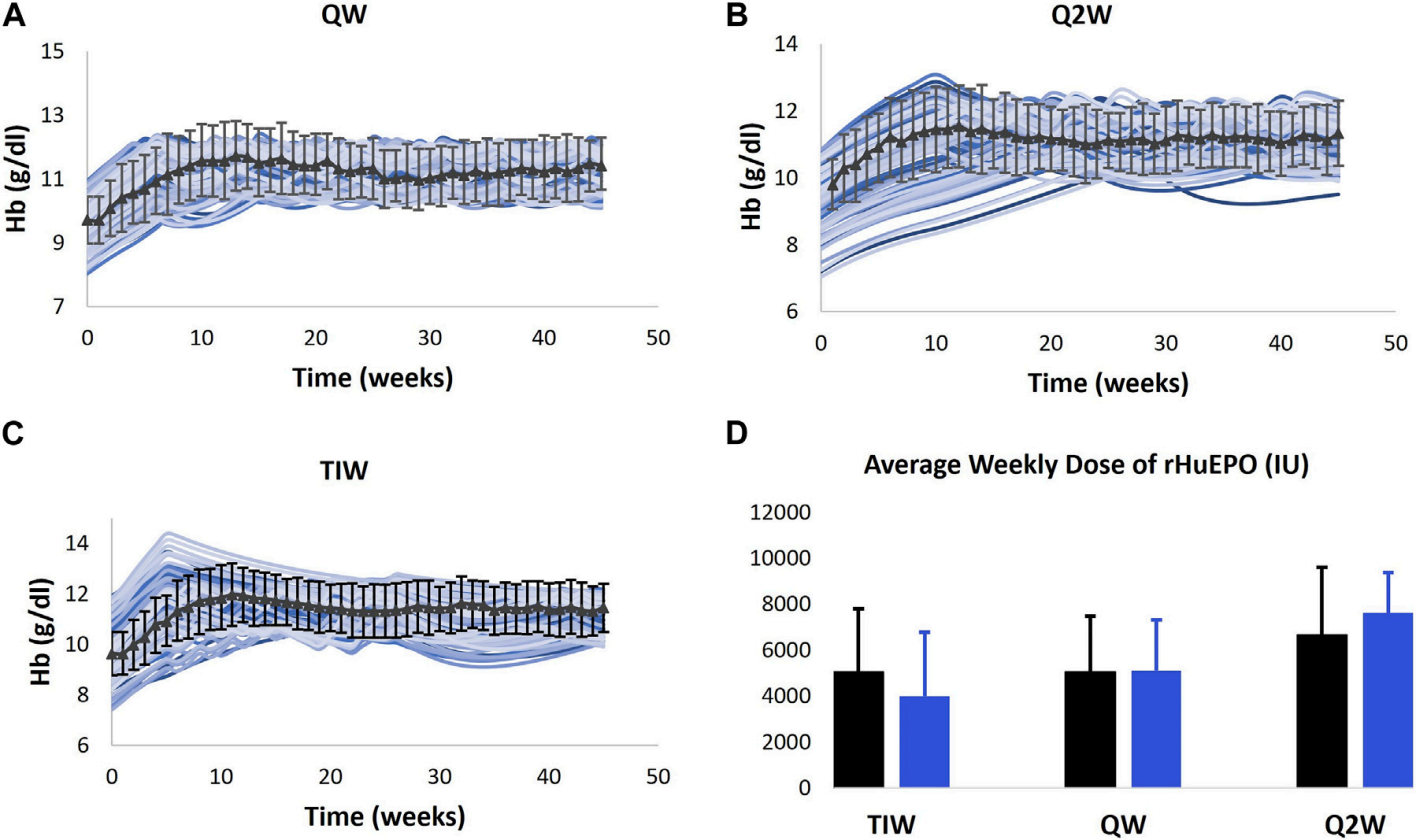
Model validation. Model prediction of Hb response to QW **(A)**, Q2W **(B)**, and TIW **(C)** dosing schedules of rHuEPO and average weekly dose **(D)** for the dosing schedules. The model is only calibrated to the Hb response to QW dosing of rHuEPO (Provenzano et al., 2004), and the validation is carried out using data obtained from Pergola et al. (2009).

Model validation is carried out by predicting scenarios not used for parameter fitting of the Vpop.

a. The model is used to predict the Hb outcomes with Q2W (biweekly) and TIW (thrice weekly) dosing of epoetin alfa compared to the calibrated QW (weekly) dosing in anemic CKD ND subjects (Pergola et al., 2009). Three groups/cohorts of CKD 3 and CKD 4 patients are created. The baseline Hb levels of Vpop in each arm spanned the corresponding Hb range reported for the trial arm in the paper. The VPs in the three arms are subcutaneously administered 50 IU/kg TIW, 10,000 IU QW, and 20,000 IU Q2W of epoetin alfa, respectively. Simulations are run using the exact titration algorithm reported in the trial. Dose increases are not allowed in the first 4 weeks. However, after the fixed dose period, dose titration is performed according to the algorithm to achieve and maintain a target Hb level of 11–11.9 g/dL. At week 23, VPs in the TIW group are switched to an initial dose of 10,000 IU QW administered subcutaneously. As shown in Figure 7, the model results are in alignment with clinical observations, which showed that the QW and Q2W dosing regimens maintain the Hb levels as efficiently as the TIW regimen. Simulations show that the mean change in the Hb level from the baseline after 44 weeks in TIW, QW, and Q2W regimens was 12.33%, 14.92%, and 17.10% compared to 18.89%, 17.54%, and 15.81% in the data, respectively.
b. For ND trials of all therapies, simulation results for the average dose administered are compared against the reported data, as shown in Supplementary Table S3 (Suranyi et al., 2003; Provenzano et al., 2004; Pergola et al., 2016; Holdstock et al., 2019). It is worth noting that the metric for reporting therapy dose is not the same across the trials. For example, for rHuEPO, the average weekly dose across the trial duration is reported, whereas for vadadustat, the average dose at the end of the trial is available. The model-predicted mean doses for rHuEPO and vadadustat Vpops are in alignment with the data. However, the median doses for darbepoetin and daprodustat Vpops did not exhibit such proximity. This can be attributed to the uncertainty associated with median dose calculations and interpretation (further details in “Validation” in the Supplementary Material).

**FIGURE 8:**
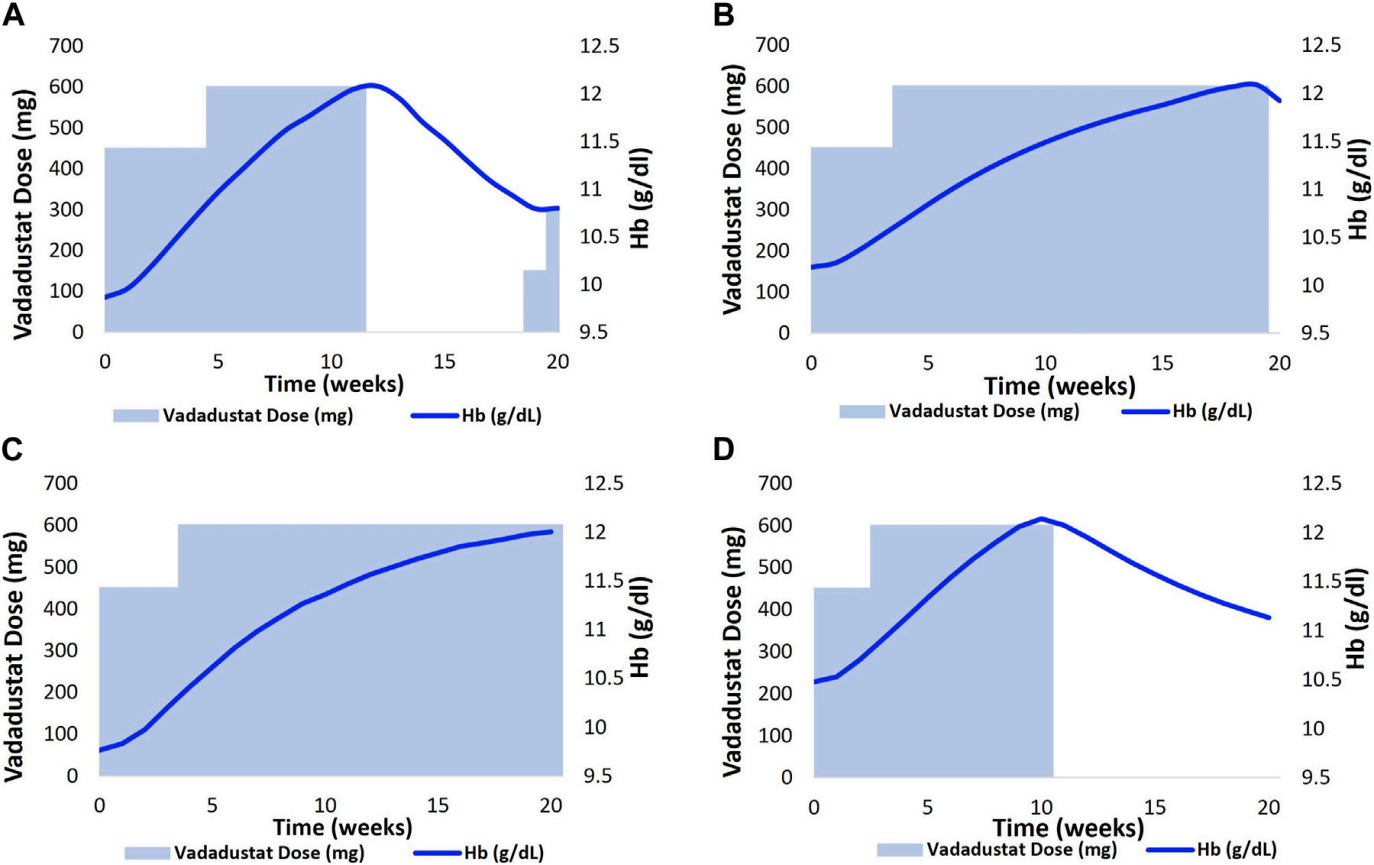
Implementation of an adaptive dosing regimen as observed in the phase 2 vadadustat clinical trial. Four VPs **(A,B)**- CKD 3; **(C)**- CKD 4 and **(D)**- CKD 5 were selected, and the Vadadustat dose adaptation protocol was applied for 20 weeks. The vadadustat dose was adjusted biweekly, based on the analysis of the Hb response. Notably, a minor modification was made to the protocol originally described by Pergola et al. (2016), wherein the dose reduction occurred as the Hb approached 12 g/dL, instead of 13 g/dL in our model.

We then aimed to visualize the dose adaptation protocol per VP in the vadadustat trial. Figure 8 represents four distinct scenarios where the dose of vadadustat has been adjusted according to the dosing algorithm (as a response to the Hb level). Each adjustment is tailor-made to the patient’s requirement, with the goal of not letting Hb reach 13 g/dL or more. (These simulations accommodate a slight modification from the protocol reported in Pergola et al. (2016) such that in the model, the dose is reduced when the Hb level approaches 12 g/dL and not when it approaches 13 g/dL (Pergola et al., 2016). The modified protocol is reported in the “Modeling Adaptive Clinical Trials”’ section.)

The upper panel corresponds to two distinct CKD 3 VPs who are being administered vadadustat at a starting dose of 450 gmg QD (Figures 8A, B). The vadadustat dose is then increased based on the patient-specific Hb response. The upper left chart shows that the dose is withheld at 75 days when it was anticipated that the Hb level will continue to increase beyond 12 g/dL. The dose is resumed when the Hb level decreases to 10.8 g/dL (Figure 8A). The bottom panel corresponds to dose titration, following the Hb response in CKD 4 and CKD 5 VPs. The CKD 4 VP required only one dose adjustment, wherein the dose was increased to 600 mg QD in the third week. Following the dose adjustment, the Hb level gradually increased to 12 g/dL (Figure 8C). The CKD 5 VP, on the other hand, followed a titration algorithm similar to CKD 3 VP in the upper left chart. However, vadadustat was not resumed during the trial after withholding the dose at day 97 (Figure 8D).

**FIGURE 9:**
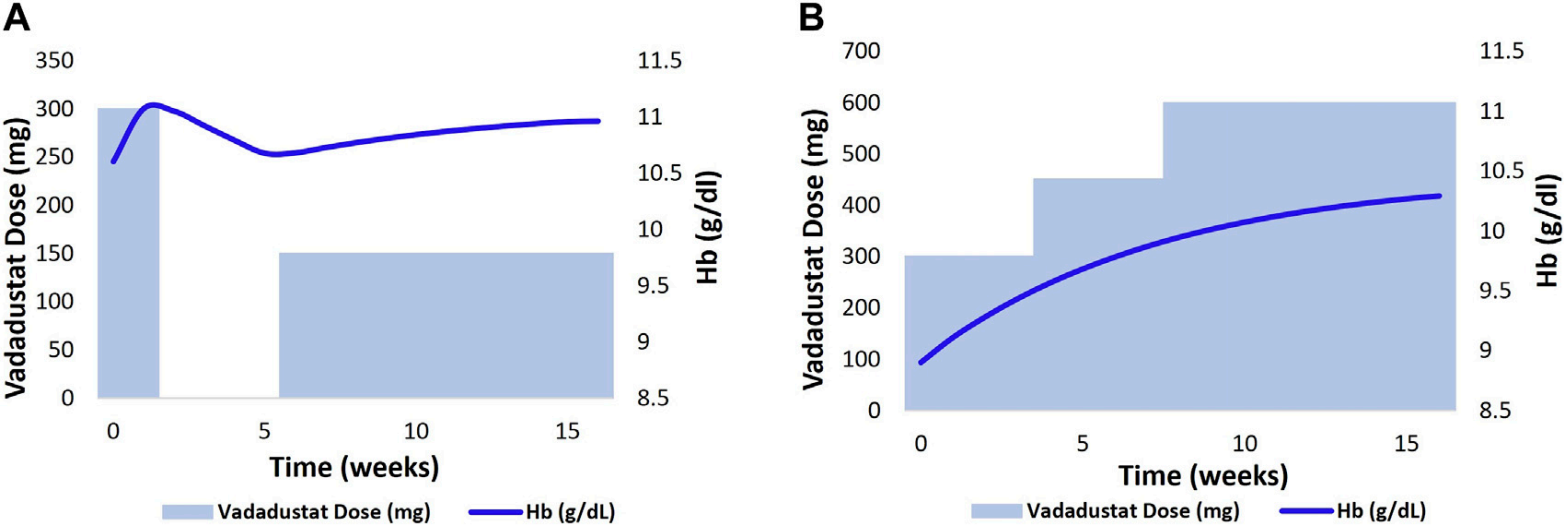
2 HD VPs (previously on ESA therapy) as shown in **(A, B)** were selected, and the Vadadustat dose adaptation protocol with target Hb level of 10–11 g/dl and starting dose of 300 mg QD was applied for a duration of 18 weeks. The adjustment of Vadadustat dose was based on the analysis of Hb response.

Figure 9 shows the Hb response to vadadustat in two HD VPs to achieve and maintain a target Hb level at 10–11 g/dL. The left figure shows the Hb trajectory corresponding to vadadustat dose adjustment in an HD VP who is on the rHuEPO dosing regimen, such that the BL Hb level reaches 10.6 g/dL. The initial dose is 300 mg QD, which causes an Hb response >11 g/dL in 2 weeks. This leads to dose interruption for 4 weeks, following which the dose is resumed at 150 mg QD, which maintains the Hb level at 11 g/dL (Figure 9A). In the right figure, the HD VP initiates treatment at a dose of 300 mg QD but needs a dose increase to 600 mg for achieving an Hb level of 10–11 g/dL (Figure 9B).

## 5 Discussion

We developed a QSP model of erythropoiesis to enable decision-making during the clinical trial stages of ESA and PHI development. We chose to model the physiology and time scales relevant to the visualization of the clinical trial results in detail (for example, Hb dynamics in a time scale of weeks) and captured implicitly physiology not directly relevant to clinical trial visualization (for example, maturation of precursors in bone marrow). The model is comprehensive in capturing population Hb response to various approved ESAs (rHuEPO, darbepoetin) and PHIs in clinical development (vadadustat and daprodustat). We also included the range of patients with CKD that are potentially benefited by ESAs, including moderately and severely affected patients who are either on hemodialysis or not on dialysis. We incorporated a key aspect of clinical trial design of ESAs and PHIs, namely, adaptive dosing to allow for differential dosing based on absolute Hb levels and change in Hb levels to maintain blood Hb levels in an optimal range such that the tradeoff between efficacy and safety is balanced (most trials aim to maintain Hb levels between 10.0 and 11.5 g/dL). The model simulations are in good agreement with the data under different conditions, which include ESA and PHI administration.

We validated this model by predicting the dose and Hb levels for multiple regimens of rHuEPO without varying the physiological parameters of the Vpop and by only varying trial design parameters. This is an important step to increase confidence in model predictions as we deploy this as a tool in supporting clinical trial design. Models are inherently limited by the data and assumptions that go into their development. To reduce the uncertainties in model predictions, models have to be calibrated to different types of data and validated against “new” or uncalibrated data. Furthermore, ranges in clinical trial predictions and recommendations based on these should be evaluated using our understanding of physiology and modeling assumptions to ensure that the model predictions are reasonable. In this case, we included multiple relevant trials and validated the model by reasonably predicting the Hb response to a different rHuEPO dosing schedule that the model was not calibrated to. Thus, model simulations enable the visualization of the individual patient trajectory over the course of the trial, and such visualization may enable supporting personalized therapy (Brier et al., 2018) evaluating compliance and generally enable reverse-translation decisions (Rostami-Hodjegan, 2018).

In recent years, phase 3 clinical trials of vadadustat and daprodustat conducted in CKD non-dialysis and dialysis patient populations have been published (Singh et al., 2021a; Singh et al., 2021b; Chertow et al., 2021; Eckardt et al., 2021). Currently, these trials are not incorporated into the model yet but may be added readily, given the basic structure and Vpops from our modeling. Daprodustat has demonstrated non-inferiority to darbepoetin alfa with respect to efficacy and safety in both populations. From the efficacy standpoint, vadadustat is also similar to darbepoetin alfa in both populations. However, vadadustat failed to meet the non-inferiority criterion for cardiovascular safety in ND patients. The clinical trial design may have impacted the ability to demonstrate non-inferiority, which may be improved by the use of our models in designing a trial (Singh, 2022).

For this new class of drugs, safety risk considerations are as important as efficacy in drug development decision-making. These insights need the mechanistic understanding of individual patient responses and may not be easily accomplished by empirical modeling of responses without explicitly considering relevant physiology. Our model focuses on efficacy in terms of Hb dynamics, and safety may be predicted if these events are correlated with Hb excursions outside the safe zone. In particular, in the case of the development of ESAs, the adaptive trial design is a major tool to limit unsafe excursions of Hb, and a tool to visualize the trade-offs in trial design can be very useful in planning future trials. In the trials comparing PHIs (daprodustat and vadadustat) against darbepoetin, PHIs were administered daily, whereas varying protocols for the administration of darbepoetin existed (Singh et al., 2021a; Chertow et al., 2021). Our model can help standardize dosing protocols in clinical trials and enable a rigorous intention-to-treat analytical approach to analyze efficacy and safety data (Singh, 2022). More work needs to be carried out to make this class of drugs as safe and efficacious as possible since they are very convenient for the ND and home dialysis patient population.

Model-informed drug development (MIDD) comprises the use of quantitative techniques to enable and increase confidence in decision-making in drug development (Wang et al., 2019). In particular, our effort is focused on developing a tool to visualize clinical trials for drugs targeting anemia due to CKD, which are complex with multiple considerations. The ways in which we can leverage this asset in clinical trials are as follows:

a. Visualization of adaptive trial design to suggest optimal dose, dosing regimen, and titration protocol for a population of ND and HD patients. The model can also be used for forward translation, i.e., predicting therapy response in a phase 3 clinical trial, given that the information on patient baseline characteristics, starting dose, and dosing regimen is provided. A Vpop capturing the variability in the participants of the trial can be generated using this information, which can then be simulated to predict the Hb behavior in response to the therapy.
b. Understanding factors about compliance, response, and other real-world considerations by reproducing a clinical trial. The model can be employed for reverse translation such that model-based scrutiny of clinical trial results provides insights into the mechanism of action of a drug and its PD effects.
c. Identification of responsive and non-responsive patients by analyzing key parametric differences between them. This future effort may be useful in understanding the underlying factors that contribute to non-responsiveness to ESA or PHI therapy, following which specific actions can be undertaken (for example, switching a patient from ESA to PHI or *vice versa* and increasing therapy dose). It can also be used to check whether factors that contribute to hypo/non-responsiveness under ND conditions are the same as those under incident HD and prevalent HD conditions.

There are several limitations to the current work that can be refined in future efforts. One key area in the improvement of Vpops is to test not only for the efficacy outcome (time course of Hb) but also for safety parameters such as the percentage of patients who have Hb excursions (Hb ≥ 13 g/dL) or delta Hb (for example, >1 g/dL increase in 2 weeks) excursions in a trial. Based on our reading, many ESA trials did not report, while some PHI trials have reported these Hb excursions explicitly in the publications. Publicly available data and proprietary data may be used to refine the Vpop and dose titration algorithm to enable better predictions. Future Vpops will also include incident and prevalent hemodialysis patients and patients undergoing peritoneal dialysis as these groups may respond differently to Hb excursions.

In the future, the scope of the model may be expanded by our team or other interested groups to include additional aspects of erythropoiesis such as Fe metabolism, hepcidin levels, and the effects of VEGF. Incorporation of iron metabolism leads to the construction of a comprehensive erythropoiesis model, which can then track the biomarkers or iron sufficiency such as ferritin, transferrin, and serum Fe measured in ESA and PHI clinical trials. We did not account for varying levels of iron stores because iron-replete stores were a key inclusion criterion for most clinical trials of rHuEPO/ESA/PHIs. All major PHI trials showed suppression of hepcidin levels with PHI, although it is not clear whether this translates into better iron mobilization and utilization (Pergola et al., 2016; Holdstock et al., 2019).

Similarly, inclusion of VEGF, which is directly regulated by HIF1, will help in tracking a key safety aspect of PHI trials (Becker and Jones, 2018). EPO increase by PHIs can affect the plasma VEGF concentration, which contributes to angiogenesis, thereby increasing the probability of the initiation/progression of cancer. All these agents, i.e., EPO, ESA, and PHIs, are associated with hypertension and major adverse cardiovascular events (MACEs) in the trials. Since blood pressure and MACEs are of serious clinical concern in this population, tracking BP responses is of considerable interest to clinicians. Incorporating population variability by considering different ethnicities, diabetes, and inflammation status is also another direction of improvement for the model. It is known that plasma volume, Hb range, and Fe metabolism vary across people of different ethnicities, and addressing this heterogeneity in the patient population is crucial to our efforts in better understanding global clinical trials (Barrera-Reyes and Tejero, 2019; Nguyen et al., 2020; Canney et al., 2023).

## 6 Conclusion

We successfully established a QSP model of erythropoiesis that replicates the Hb dynamics in response to ESAs and PHIs in different types of CKD patients. We demonstrated how the model can handle an adaptive trial design protocol and reported different ways in which the model can be employed to support decision-making in clinical drug development.

## Data Availability

The original contributions presented in the study are included in the article/Supplementary Material; further inquiries can be directed to the corresponding author.
