# Supplementary material for: Quantitative systems pharmacology model of erythropoiesis to simulate therapies targeting anemia due to chronic kidney disease
Source: Front Pharmacol. 2023 Dec 6;14:1274490. doi: 10.3389/fphar.2023.1274490 (PMC10731587; doi:10.3389/fphar.2023.1274490)

**Define U in Simbiology**

To upload and run the model, one has to create a unit called U in simbiology. The U is the unit for EPO where EPO is measured in mIU/ml or U/L.

U is related to nanogram as follows


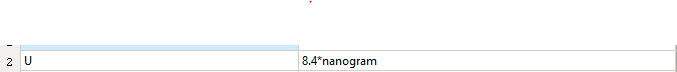


A snapshot of U in the simbiology library is also shown below


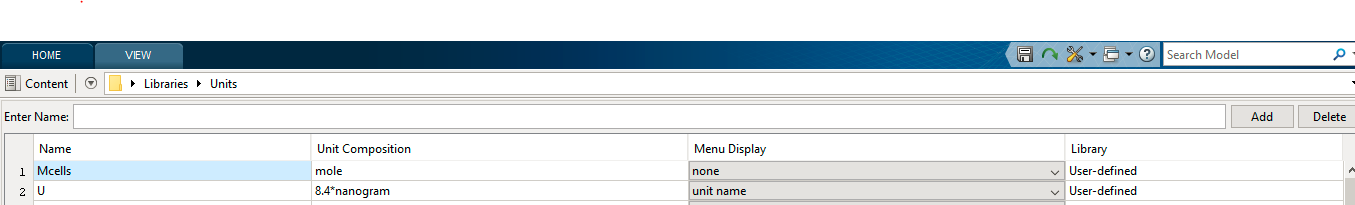

Supplement: Supplementary file 1 [file DataSheet1.ZIP › Model File and Scripts/Readme.docx]
