# Supplementary material for: Quantitative systems pharmacology model of erythropoiesis to simulate therapies targeting anemia due to chronic kidney disease
Source: Front Pharmacol. 2023 Dec 6;14:1274490. doi: 10.3389/fphar.2023.1274490 (PMC10731587; doi:10.3389/fphar.2023.1274490)
Supplement: Supplementary file 2 [file Table2.DOCX]

### Supplementary Appendix

###

###

###

###

###

###

###

###

###

###

###

###

###

### Mathematical Models of Erythropoiesis in the Literature

## Mechanistic models of Erythropoiesis where PHD-HIF pathway is not explicitly captured

Previously, age-structured models of erythropoiesis were fairly common as the one developed by Belair et al., 1995 and were used to analyze EPO and Hb dynamics in response to various perturbations like blood donation, chemotherapy, ESA administration, etc. Age structured models divide erythroid progenitor cells into subsets based on maturation stage. Cell population in each stage depends on its immediate precursors in the previous time step. This model showed that a perturbation of the erythropoietic system following a blood donation event in normal humans leads to a damped oscillatory return of the system to normal [Belair et al., 1995].

An age-structured model of erythropoiesis in humans that uses a system of partial differential equations to study the variable velocity of aging of precursor cells was published by Mahaffy and co-workers [Mahaffy et al., 1998; Mahaffy et al., 1999]. Addition of an EPO-dependent rate of apoptosis of precursor cells and introduction of a moving boundary condition for mature erythrocytes provides a variable maximal lifespan of RBC in this work. Loeffler and colleagues have developed a mathematical model that is primarily based on the assumption that the number of cell divisions taking place in erythropoietic precursor stages is primarily regulated by EPO depending on the oxygen supply to the tissue [Loeffler et al., 1989]. This model reproduces data obtained in situations of stimulated erythropoiesis (changes in tissue oxygen tension due to bleeding, hemolytic anemia) or increase in plasma volume or decrease in atmospheric oxygen pressure (hypoxia)) in mice and rats [Wichman et al., 1989]. Studies to evaluate the effect of various conditions on erythropoiesis like benzene intoxication, changes in the altitude of residence, administration of ESAs to patients prior to elective surgeries have also been undertaken using age-structured models [Banks et al.,2004; Fuertinger et al., 2012].

Schirm et al.,2013 developed a cell kinetic model of bone marrow erythropoiesis and its regulation by endogenous EPO which simulates physiological response to EPO and chemotherapy administration. [Schirm et al., 2013]. It describes the proliferation and maturation of erythropoietic cell stages, with EPO mediated feedback loop regulating the erythropoietic process. Chemotherapy is modeled by a reversible and transient depletion of precursor cells; with delay and toxicity parameters incorporated to capture the maximal damage of chemotherapy. A PK model of EPO proposed by Krzyzanski et al.,2007 and a sheep EPO injection model proposed by Kota et al., 2007 were integrated to the cell kinetic model by Schirm and team to account for the effects of external EPO administrations [Krzyzanski et al., 2007; Kota et al.,2007]. Later, the scope of the model was extended by incorporation of an iron metabolism sub-model regulated by hepcidin [Schirm and Scholz, 2020]. This model captures the physiological mechanisms of iron homeostasis including storage iron, plasma transferrin, non-transferrin bound iron, iron stored in cells of the red blood cell lineage and iron in enterocytes. The model is linked to the former erythropoiesis model by Schirm by affecting hemoglobinization of RBC precursors and considering the recycling of iron by the hemoglobin catabolic system. The model can explain time courses of RBCs, reticulocytes, Hb, hematocrit, RBCs, EPO, serum iron, ferritin, transferrin saturation, and transferrin following EPO and iron application into healthy volunteers or chemotherapy patients. Furthermore, the authors also constructed a hybrid model by combining the above mentioned erythropoiesis model with a cell kinetic model of granulopoiesis (inhouse) with a common stem cell model so as to study the dynamics of mature blood cells and cytokines under conventional chemotherapy, G-CSF and EPO applications [Schirm et al., 2014].

## Erythropoiesis Models with emphasis on rHuEPO PK

Numerous PK models depicting the serum EPO concentration after ESA administration exist in the public literature. A PK/PD model of EPO in sheep was developed by Veng-Pederson et al., 2002 and Chapel et al.,2000 [Veng-Pederson et al., 2002; Chapel et al., 2000]. Ramakrishnan et al.,2004 developed a PK/PD model to account for rHuEPO concentrations in healthy volunteers following intravenous and subcutaneous dosing [Ramakrishnan et al., 2004]. The model incorporates a cell production and loss mechanism with a feedback down regulation of endogenous EPO secretion to capture the pharmacodynamics effects of SC rHuEPO dosing and downstream effects on reticulocytes, RBC, and Hb. They reported that a one compartment model with limited distribution and nonlinear elimination was adequate to characterize the data from the IV dosing. A dual-absorption rate model (fast zero-order and slow first-order inputs) with nonlinear disposition characterized the PK of SC rHuEPO. This PK model structure formed the basis of the one employed by Krzyanski et al.,2005 who used linear disposition kinetics (as opposed to non-linear) [Krzyanksi et al., 2005].

A mechanistic PK/PD model was developed by Woo et al.,2007 to describe rHuEpo PK characteristics following IV administration over a large range of single doses in humans (also parameterized for rats and monkeys) [Woo et al.,2007]. Target mediated drug disposition (TMDD) following EPO binding to EPOR was established as a primary mechanism for nonlinear clearance of rHuEPO as well as activation of erythropoietic precursors. The PK model components include receptor binding, subsequent internalization and degradation, EPOR turnover, non-specific tissue distribution, and linear first-order elimination from plasma whereas the PD model includes a feedback mechanism by which the increase in Hb in circulation inhibits further stimulation of progenitor production in bone marrow. Additionally, the EPO–EPOR complex was applied to describe erythropoietic effects of rHuEPO in rats.

## Mechanistic models of Erythropoiesis with PHD-HIF pathway

Singh et al.,2015 developed a systems pharmacology model of erythropoiesis in mice by incorporating all major components of the PHD2-HIF1α pathway that control the erythropoietic response [Singh et al., 2015]. A systems approach was utilized to integrate intracellular HIF1α and EPO mRNA responses with downstream in-vivo biomarkers of erythropoiesis such as EPO plasma levels, reticulocyte counts, RBC, and Hb. The model is able to simulate Hb and RBC dynamics following PHI administration.

# Model Design and Equations

## Model Design Considerations

Conceptually, the dynamics of the model occurs in 4 different compartments - Kidney, Bone Marrow (BM), Plasma, Peripheral (Figure 1, main text). However, apart from peripheral, the other 3 compartments are not modeled as separate compartments in Simbiology. In particular, the EPO_plasma signals to the progenitors in the BM and all the species are represented within a single compartment, referred to as 'Central.' The quantitative relationships between Hb and PHD, PHD and HIF, HIF and EPO and EPO & RBC for a particular Virtual Patient (CKD Ref VP3) are shown in Figure S2 to give an understanding of the model dynamics.

In other estimates, the BM volume and Plasma Volume though not identical, are comparable(BM ~ 0.5x plasma volume) [Nombela-Arrieta and Manz,2017].. Progenitors, Precursors and the interaction between EPO and EPO-Receptors is quantified in terms of Cells/Volume, making the compartment's volume a critical factor influencing the concentrations of these species. Since we have simplified this dynamics as occurring in the same compartment i.e plasma, the model simulations on Progenitors and Precursors numbers, both of which exist in the BM may be unreliable due to the fact that the volume of the Bone Marrow is likely smaller than that of the Central compartment. To improve the model's accuracy in predicting Progenitors and Precursors numbers, an enhanced approach involves incorporating a separate compartment specifically for the Bone marrow in the model. However, the beginning (EPO plasma) and end (Retics plasma) are consistent with reported data making that simulation reliable.

*
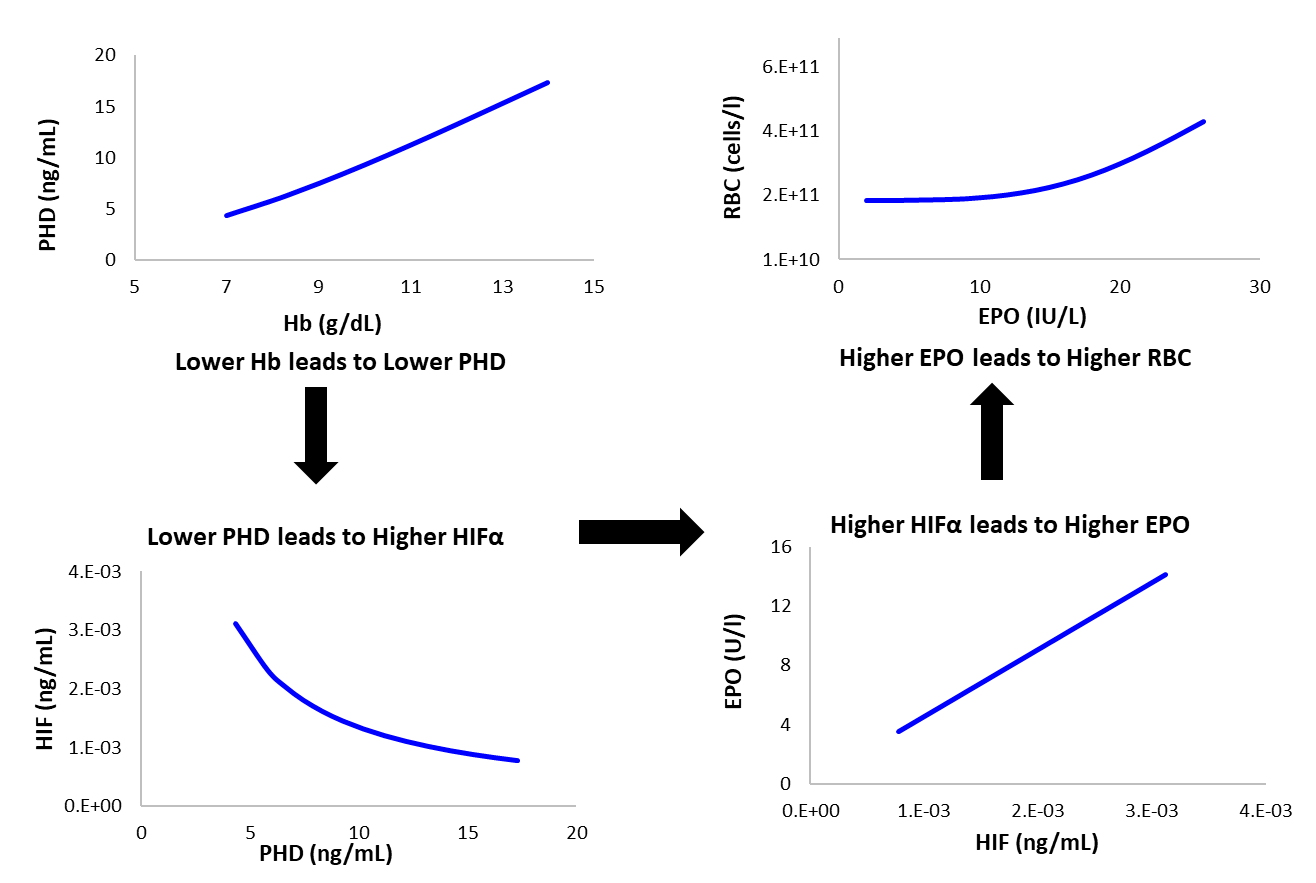
*

**Figure S1:** This Figure shows the quantitative (approximate, assuming steady state receptor-ligand interactions & peripheral-central flux) relationships in the key feedback loop in the model. The top left figure shows the relationship between Plasma Hb and PHD in the kidney in the model. Here while Hb is calibrated to data, PHD & HIF in the kidney have not been calibrated explicitly to data and are heuristics in the model. The bottom left shows HIF increasing as PHD decreases. The bottom right shows plasma EPO increasing as HIF in the kidney increases. This relationship is dependent on the disease severity as patients with better secretion capability respond more strongly (based on EPO production rate which varies with disease severity). The quantitative relationship is shown for CKD 3 VP. The top right shows the increase in RBC as EPO increases, also for CKD 3 VP.

## Detailed Equations

All equations are laid out in the main text. Some equations are expanded here to enable ease and clarity of reading in the main and Supplementary sections.

The PHD concentration is modeled as a function of PHI and Hb, as shown in Equation [i], in the main text, is expanded here

*PHD = PHD_basal/(1+PHI_effect+Hb_effect)*

*=PHD_basal/(1+Vmax*PHI_drug_conc^n1/(Km^n1+PHI_drug_conc^n1)+ k_Hb_effect_on_PHD/(Km+Hb^n2)*

The rate of degradation of the progenitors as regulated by EPO-EPOR is shown as a function of EPO-EPOR concentration in Equation YY in the main text. That, in fact, is a higher order (n=4) Hill function as shown below.

***Rate of degradation of progenitors regulated by EPO-EPOR*** *= k_baseline_deg*Progenitors*(1-(([Vmax_EPO-EPOR_effective]*((EPO_LR_complex))^[nEPO-EPOR_to_Progenitors])/((*[Km_EPO-EPOR_Progenitors])^[nEPO-EPOR_to_Progenitors]+(scalar_EPO_efective*(EPO_LR_complex))^[nEPO-EPOR_to_Progenitors])))*

The interaction between the EPO and EPO receptor takes place in molecule/volume units, as defined by the equation provided below. EPO_plasma in the equation is in the units of molecule/mL. The conversion of rHuEPO_plasma from ng/mL to molecule/mL is as follows:

Molar weight of EPO= 30,000 (g/mol) [Cheung et al., 2001]

Molecular weight of EPO= 30,000 (g/mol)/ (6.022e+23 molecules/mol) = 5e+11 ng/molecule

EPO_plasma_molecule_mL = EPO_plasma(ng/mL)/(5e+11 (ng/molecule))

*d(EPO_LR_complex)/dt= ((kon_EPO_LR_complex * EPO_receptor * EPO_plasma_molecule_mL) - (koff_EPO_LR_complex * EPO_LR_complex)) - (kdeg_EPO_LR*EPO_LR_complex)*

## Developing Virtual Patients

The following parameters are varied from “baseline” to create the Healthy Virtual Patient and the CKD patients of various stages based on our best understanding of the pathophysiology of anemia in CKD.


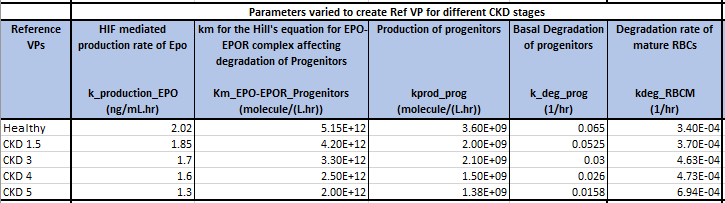


**Table S1:** Parameters that are varied to create Healthy and CKD Ref VPs of increasing severity.

In particular, with increasing pathophysiology of the disease, there is decrease in HIF mediated production of EPO, increase in the degradation rate of progenitors, RBCs and decrease in the production rate of progenitors. We believe the “km for the Hill's equation of EPO-EPOR complex affecting degradation of Progenitors” may need to increase with increased disease severity, however, the constraints that we had set up were inadequate to ensure that behavior. This may be enforced as an additional constraint in future efforts.

In order to ensure response to therapy, 2 Reference VPs - ND & HD are created from which the VPop is generated (Figure S2 and S3).

Following rHuEPO calibration using healthy Ref VP, we then proceeded with simulations of diseased conditions. A CKD4 VP is selected to represent ND VP as it is observed that ND trials have a higher percentage of CKD 4 (>50%) patients than CKD 3. A single ND and ESA naive VP of CKD stage 4 is calibrated to both untreated baseline clinical read-outs (Hb, EPO) and Hb response to rHuEPO, darbepoetin, vadadustat and daprodustat reported in clinical trials of interest [Provenzano et al., 2004; Suranyi et al., 2003; Pergola et al., 2016, Holdstock et al.,2016].

Figure S2 shows that the model fits for ND are in good agreement with the mean/median Hb response of the trial population. Over the course of 16 weeks, 10000 IU QW rHuEPO is able to raise the Hb level by 2.9g/dl in a ND Ref VP. Similarly, ND Ref VP with 0.75 µg/kg of Darbepoetin once every other week (Q2W) for 24 weeks have their baseline Hb elevated by 2.6g/dl. On the other hand, 450 mg of Vadadustat QD is responsible for an increase of baseline Hb by 1.32g/dl at the end of 19 weeks of dosing and 4 µg of Daprodustat QD for an increase of baseline Hb by 1.4g/dl at the end of 25 weeks of dosing in an ND Ref VP. Therefore, the VP response to rHuEPO, Darbepoetin, Vadadustat and Daprodustat of doses 10000 IU QW, 55 μg Q2W, 450 mg QD and 4 µg QD respectively is consistent with what is reported in the trials.


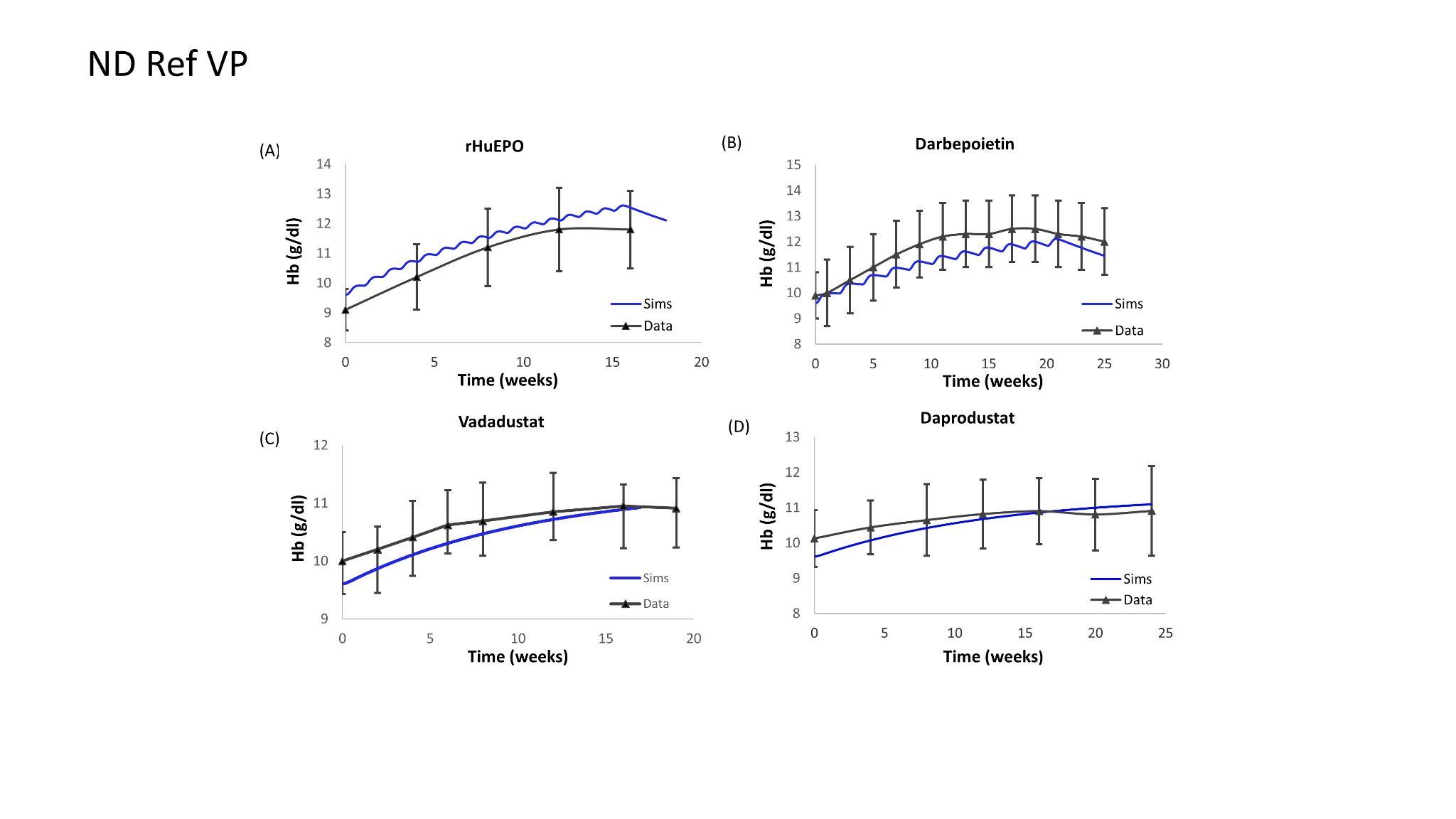


**Fig S2: Comparison of model and data for Hb response following therapy in a ND Ref VP.** Simulations are run on a CKD 4, ESA naive ND Ref VP following initiation of either rHuEPO, Darbepoetin, Vadadustat and Daprodustat. The model is then calibrated with the Hb data obtained from clinical trials of the therapies. Blue lines correspond to model output for a Ref VP. Black solid line with markers represents clinical data in the trials of interest. rHuEPO, Darbepoetin and Daprodustat data are reported as mean + standard deviation whereas Vadadustat data is reported as median, 25th percentile and 75th percentile. Note that the ND Ref VP was optimized independently of Daprodustat data and was subjected to Daprodustat only after the completion of the entire model calibration process, as illustrated in (D).

A CKD5 VP is selected to represent HD VP as >90% of patients in HD trials are of CKD 5 who have been treated with rHuEPO for months and still exhibit anemia. The BL characteristics of these VPs are within ranges reported in trials recruiting HD patients [Nissenson et al., 2002; Locatelli et al., 2003; Haase et al., 2019; Meadowcroft et al., 2018]. Similar to conditions in typical hemodialysis clinical trials, these VPs were initially treated with rHuEPO for 3-4 months during which Hb reaches steady state (and reported as baseline characteristics for the trial for the novel anti-anemia agent). This is followed by 1-2 weeks of washout after which the treatment of interest (Darbepoetin, Vadadustat or Daprodustat) is administered for a specified duration. As depicted in Fig S3, rHuEPO achieved a Hb level of 11.3g/dL. It is seen that during the course of the trial, Darbepoetin maintained the Hb at 11.3g/dl which was the baseline achieved by rHuEPO dosing. Similarly Vadadustat maintained the Hb at 10.5g/dl and Daprodustat showed a slight increase in Hb from 10.3g/dL to 10.8g/dl.

In the case of rHuEPO and Darbepoetin, the HD Ref VPs match the ESA trial results reasonably well. However, it is observed that for both the PHIs, the model fit to the Hb response at later stages of the trial is not as good as that obtained with ESAs, although the fits fall within the error bars. The difficulty in matching the data may stem from the lack of information of the dose titration protocol that was frequently observed in the clinical trial. Lack of information on the trial design, specifically on previous ESA treatment and washout period led us to evaluate the impact of different washout periods on maintenance of a stable Hb level. To achieve this, a CKD 5 Ref VP (with baseline Hb level of 9.5g/dl) was administered rHuEPO QW until the Hb concentration reached 10.5g/dl. rHuEPO administration was then stopped and the Ref VP was switched to therapy of interest and the ability of the new therapy to maintain Hb level was observed. In Fig S3 (B) and (C), simulations for 3 different scenarios are presented, each with different washout duration (ranging from 0-14 days). It is observed that Hb conc falls proportional to increase in the washout duration before rising to the level seen in Pre-Vadadustat period. The therapy then acts to increase the Hb level to what was observed before the washout period.


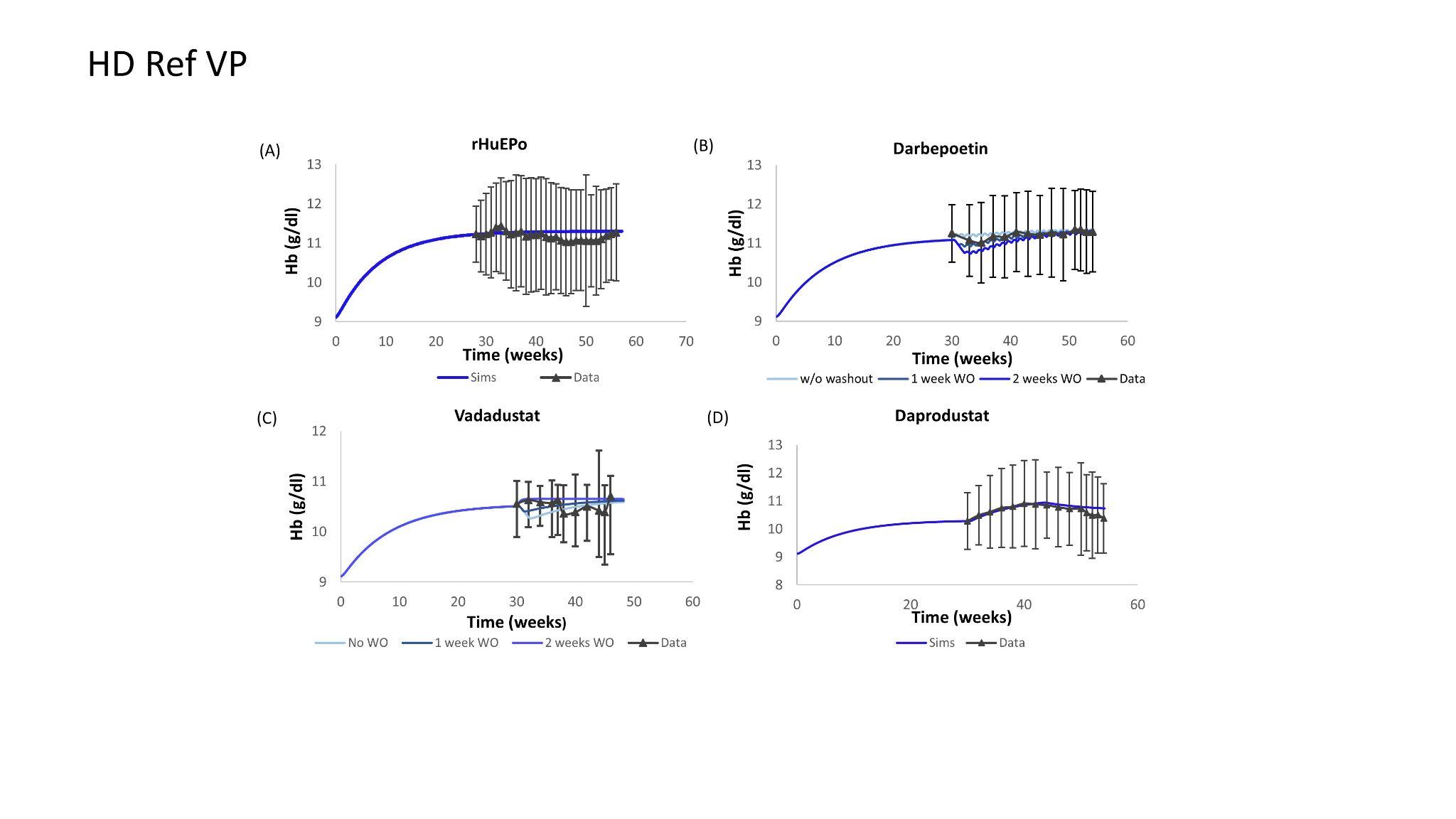


**Fig S3: Comparison of model and data for Hb response following therapy. In a HD Ref VP.** Simulations are run on a CKD 5, Ref VP who has been treated with rHuEpo previously. The model is then calibrated with the Hb data obtained from clinical trials of the therapies (rHuEPO, Darbepoetin, Vadadustat and Daprodustat). Blue lines correspond to model output for a Ref VP. Black solid line represents clinical data in the trials of interest. rHuEPO, Darbepoetin and Daprodustat data are reported as mean + standard deviation whereas Vadadustat data is reported as median, 25th percentile and 75th percentile. Darbepoetin and Vadadustat charts also show how Hb level falls from rHuEPO induced baseline value with progressive increase in washout period (from 0-14 days). WO- Washout

# Developing Virtual Populations

A distinct combination of parameter values allows each member of a population to respond to an identical dose of drug in a distinct way which eventually results in a distribution of responses that we typically observe in trials. The time dynamics data of Hb obtained from these clinical trials were used to parameterize VPop behavior.


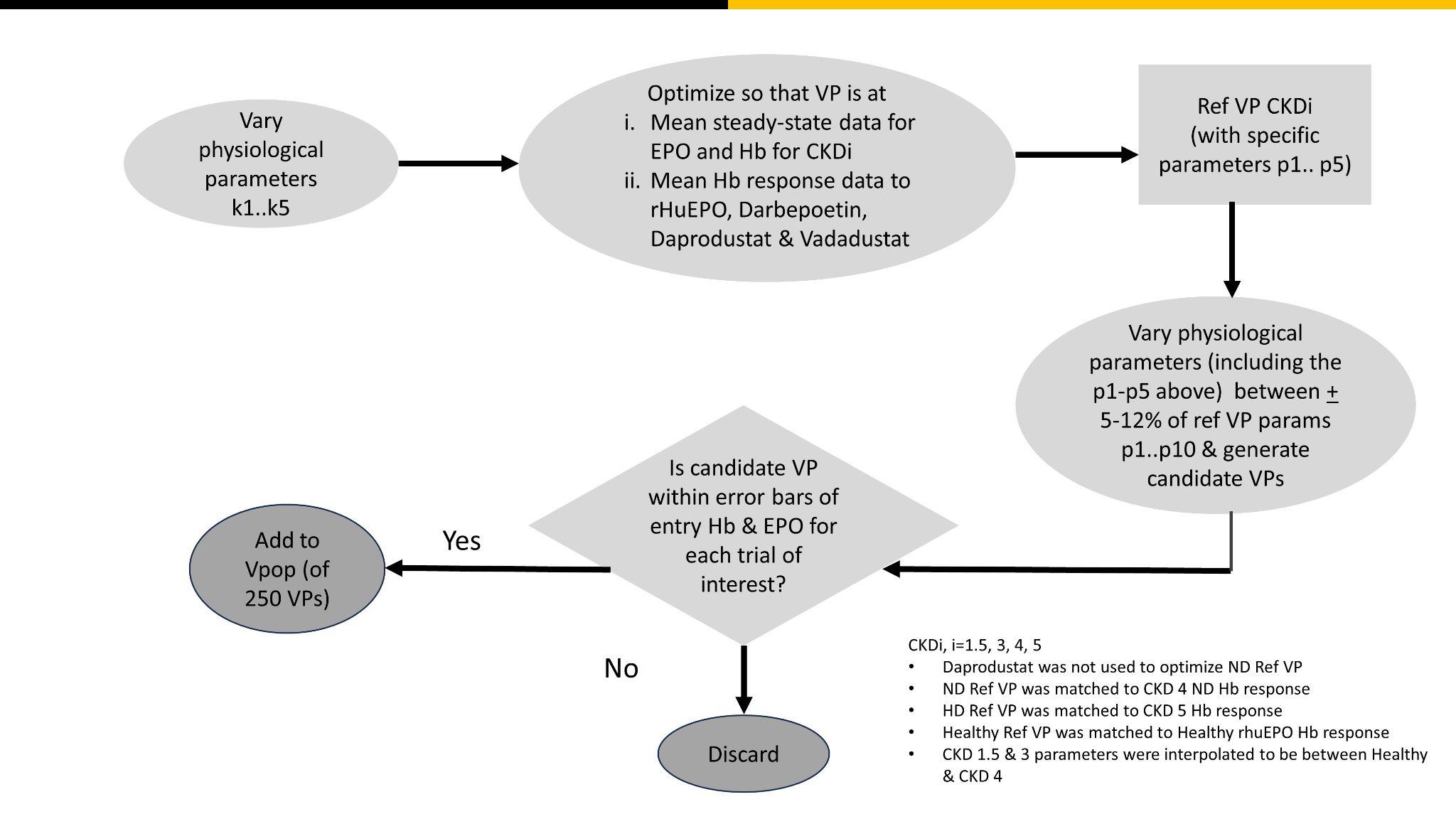


**Figure S4:** Workflow of developing Virtual Population from the Ref VP

The Vpop represents the population reported in the trials of interest. These trials are for rHuEPO (Baseline Hb: 5.9-10.0 mg/dl), Darbepoetin alfa (Baseline Hb: 9.0-10.8 mg/dl), Vadadustat (Baseline Hb: 9.04- 10.8 mg/dl) and Daprodustat (Baseline Hb: 8.0-11.0 mg/dl). These 4 ND Vpops corresponding to the three trials had the following distribution of CKD 3, 4 and 5 Ref VPs. In the case of rHuEPO, it is 10% CKD 3, 60% CKD 4 and 30% CKD 5. For Darbepoetin, it is 65% CKD 4 and 35% CKD 5 and for Vadadustat, it is 26% CKD 3, 61% CKD 4 and 12% CKD 5, Parameters were varied across a specified range around the ND Ref VP to introduce variability in the population consistent with disease severity of CKD 3, 4 and 5 estimated for the reference VPs. Cohorts are then created by filtering subjects whose steady state Hb and treatment response falls within the BL range reported in the trial. Similarly, 4 different HD-Vpops consisting of previously rHuEPO treated CKD 5 patients were created representing the population of rHuEPO, darbepoetin, Vadadustat and Daprodustat.

The following steps were taken to create a Vpop:

- Selection of a published clinical trial specific to a therapy and documenting the mean and standard deviation of the population response (in terms of Hb dynamics) as reported in the trial.
- Identification of relevant parameters that capture all the major sources of variability in the model state variables. These are motivated by sensitivity analyses as well as based on expert understanding of mechanistic causes of disease variability. In the model, parameters such as HIF mediated EPO production rate, RBC degradation rate were varied to create the Vpop and the ranges were determined by data for the physiologically accepted boundary for each parameter. Log-normal distribution was assumed while randomly drawing values for each of the parameters to create one virtual patient. Log-normal distribution was used as there are ample studies which report that distribution of biological entities such as cellular proteins follow a log-normal distribution [Eden et al., 2011; Spencer et al., 2009; Sigal et al., 2006].
- Generation of multiple plausible Virtual Patients (n=10000) and finalization of the Vpop by filtering subjects (n=300 per treatment) whose steady state Hb falls within the baseline range reported in the trial.
- Simulation of the cohort with therapy of interest, for instance rHuEPO, by following protocol reported in the trial as closely as possible. The starting dose and the protocol for dose titration as reported in the clinical trials of rHuEPO, darbepoetin, Vadadustat and Daprodustat have been replicated in the Vpop.
- Simulation of the adaptive dosing protocol to match the clinical endpoint data (This may require reasonable adjustments for details of the trials that are not explicit, for example, we observed for Vadadustat, terminating a dose when Hb = 12 g/dl instead of Hb = 13g/dl that is reported in the trial results in a better match to population data and is consistent with clinical practice).

Following therapy initiation followed by dose titration in the course of the trial, the model was able to capture the variability in Hb behavior (as a response to therapy) in a reasonable manner. The model output i.e blood Hb level for each Vpop lies within the ranges reported in the trial as shown in Figure 5 and Figure 6.

The following table lists the sensitive parameters and the percentage by which they are varied to generate Virtual Populations of ND and HD CKD trials.


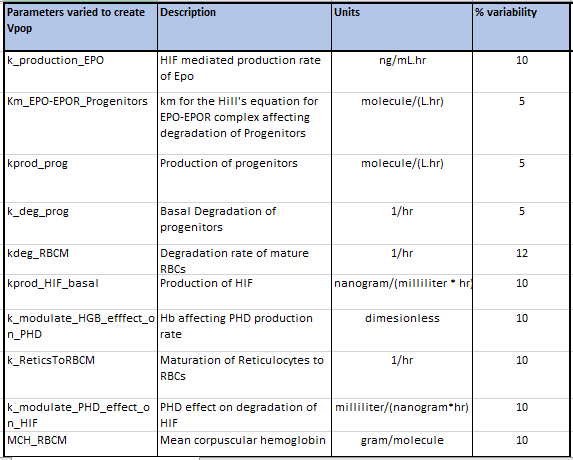


**Table S2:** Sensitive parameters and the percentage by which they are varied to generate Virtual Populations of ND and HD CKD trials. Note that 5 additional parameters are varied to create Vpop as compared to only 5 for Ref VPs.

# Validation


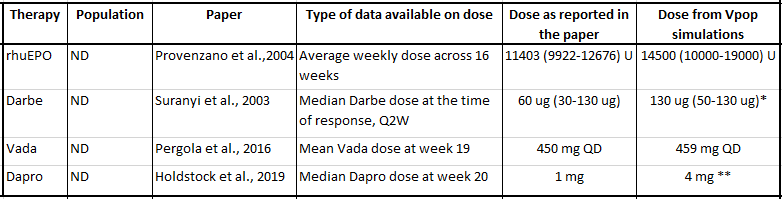


**Table S3:** Model results vs data comparison of dose for different therapies. See below for details of dose estimated for Darbe (*)and Dapro (**).

The mean dose values for rHuEPO and Vada from the model (14500 IU and 459 mg respectively) are in reasonable agreement with mean dose values reported in the rHuEPO and Vadadustat trials (11403 IU and 450 mg respectively) [Provenzano et al., 2004; Pergola et al., 2016]. However, this is not true for median dose values for Darbepoetin and Daprodustat.

*The data reported for Darbepoetin, i.e., “Median Darbepoetin dose at the time of response” is not well defined in the paper [Suranyi et al., 2003]. The corresponding dose value from the model has been calculated by taking the median of doses for individuals at which the Hb >11.0 was reached for the first time, as the target Hb range reported in the paper is 11.0-13.0 g/dL. Therefore, the model estimated dose can’t be compared with the dose reported in the paper.

**In the case of Daprodustat trial, the therapy arm with rHuEPO naive patients is divided into 2 cohorts (USA and ex-USA which differed based on BL Hb entry criteria and target Hb level) [Holdstock et al., 2016] and the trial reports the median Daprodustat dose for the combined cohorts. This limits our ability to compare the model results with that of data as only cohort 2 (ex-USA with BL Hb range of 8-11g/dl and target Hb level of 10-11.5 g/dl) data has been used for Vpop generation and the simulation results represent the median dose for this specific subset.


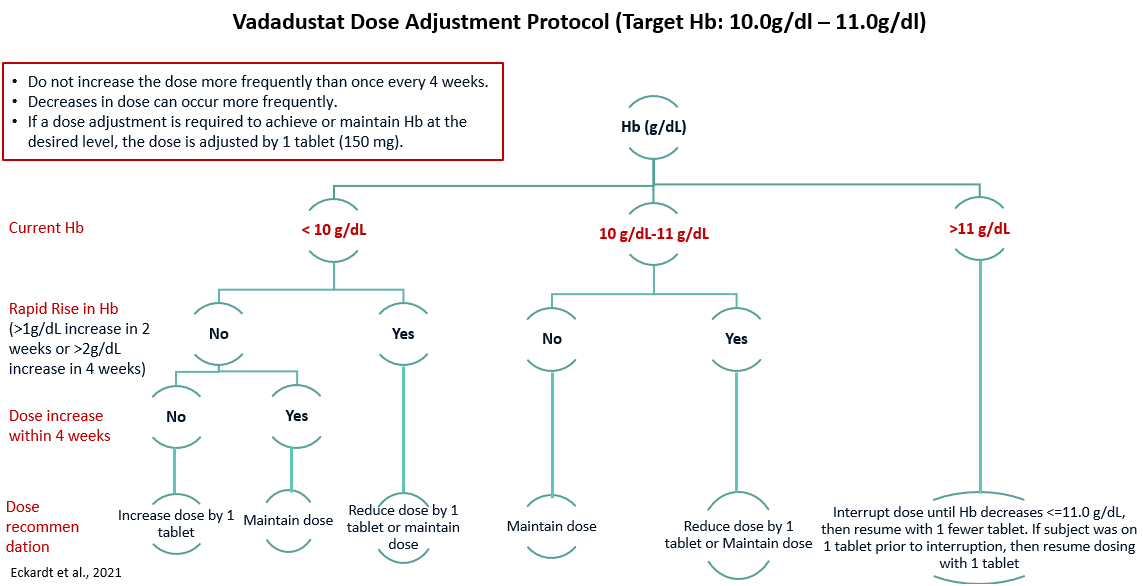


**Figure S5:** Vadadustat Dose Adjustment Protocol based on target Hb level of 10-11g/dl in Phase 3 Hemodialysis Clinical Trial (Eckardt et al., 2021)

**REFERENCES**

1. Bélair, J., Mackey, M. C. and Mahaffy, J. M. (1995) ‘Age-structured and two-delay models for erythropoiesis’, *Mathematical biosciences*. United States, 128(1–2), pp. 317–346. doi: 10.1016/0025-5564(94)00078-e.
2. Mahaffy, J. M., Bélair, J. and Mackey, M. C. (1998) ‘Hematopoietic model with moving boundary condition and state dependent delay: applications in erythropoiesis’, *Journal of theoretical biology*. England, 190(2), pp. 135–146. doi: 10.1006/jtbi.1997.0537.
3. Mahaffy, J. M., Polk, S. W., Roeder, R. K., (1999) ‘An age-structured model for erythropoiesis following a phlebotomy’. *Tech. Rep. CRM-2598,* Department of Mathematical Sciences, San Diego State University, San Diego, CA 92182-0314 .
4. Loeffler, M. Pantel K, Wulff H, Wichmann H. (1989) ‘A mathematical model of erythropoiesis in mice and rats. Part 1: Structure of the model’, *Cell and tissue kinetics*. England, 22(1), pp. 13–30. doi: 10.1111/j.1365-2184.1989.tb00198.x.
5. Wichmann, H. E. Loeffler M, Pantel K, Wulff H. (1989) ‘A mathematical model of erythropoiesis in mice and rats. Part 2: Stimulated erythropoiesis’, *Cell and tissue kinetics*. England, 22(1), pp. 31–49. doi: 10.1111/j.1365-2184.1989.tb00199.x.
6. Banks, H. T. *et al.* (2004) ‘Modeling and optimal regulation of erythropoiesis subject to benzene intoxication’, *Mathematical biosciences and engineering : MBE*. United States, 1(1), pp. 15–48. doi: 10.3934/mbe.2004.1.15.
7. Fuertinger, D. H., Kappel F, Thijssen S, Levin NW, Kotanko P. *.* (2012) ‘A model of erythropoiesis in adults with sufficient iron availability’, *Journal of mathematical biology*. Germany, 66(6), pp. 1209–1240. doi: 10.1007/s00285-012-0530-0.
8. Schirm, S., Engel C, Loeffler M, Scholz(2013) ‘A biomathematical model of human erythropoiesis under erythropoietin and chemotherapy administration’, *PloS one*. United States, 8(6), p. e65630. doi: 10.1371/journal.pone.0065630.
9. Kota, J., Machavaram KK, McLennan DN, Edwards GA, Porter CJ, Charman SA. (2007) ‘Lymphatic absorption of subcutaneously administered proteins: influence of different injection sites on the absorption of darbepoetin alfa using a sheep model’, *Drug metabolism and disposition: the biological fate of chemicals*. United States, 35(12), pp. 2211–2217. doi: 10.1124/dmd.107.015669
10. Krzyzanski, W. and Wyska, E. (2007) ‘Pharmacokinetics and pharmacodynamics of erythropoietin receptor in healthy volunteers’, *Naunyn-Schmiedeberg’s archives of pharmacology*. Germany, 377(4–6), pp. 637–645. doi: 10.1007/s00210-007-0225-z.
11. Schirm, S. and Scholz, M. (2020) ‘A biomathematical model of human erythropoiesis and iron metabolism’, *Scientific reports*. England, 10(1), p. 8602. doi: 10.1038/s41598-020-65313-5.
12. Schirm, S., Engel C, Loeffler M, Scholz M (2014) ‘A combined model of human erythropoiesis and granulopoiesis under growth factor and chemotherapy treatment’, *Theoretical biology & medical modelling*. England, 11, p. 24. doi: 10.1186/1742-4682-11-24.
13. Veng-Pedersen, P. Chapel S, Schmidt P, Al-Huniti N, Cook R, Widness J. (2002) ‘An integrated pharmacodynamic analysis of erythropoietin, reticulocyte, and hemoglobin responses in acute anemia’, *Pharmaceutical research*. United States, 19(11), pp. 1630–1635. doi: 10.1023/a:1020797110836.
14. Chapel, S. H. Veng-Pedersen P, Schmidt RL, Widness JA. (2000) ‘A pharmacodynamic analysis of erythropoietin-stimulated reticulocyte response in phlebotomized sheep’, *The Journal of pharmacology and experimental therapeutics*. United States, 295(1), pp. 346–351. Available at: https://www.ncbi.nlm.nih.gov/pubmed/10992000.
15. Ramakrishnan, R. , Cheung WK, Wacholtz MC, Minton N, Jusko WJ. (2004) ‘Pharmacokinetic and pharmacodynamic modeling of recombinant human erythropoietin after single and multiple doses in healthy volunteers’, *Journal of clinical pharmacology*. England, 44(9), pp. 991–1002. doi: 10.1177/0091270004268411.
16. Krzyzanski, W. Jusko WJ, Wacholtz MC, Minton N, Cheung WK.(2005) ‘Pharmacokinetic and pharmacodynamic modeling of recombinant human erythropoietin after multiple subcutaneous doses in healthy subjects’, *European journal of pharmaceutical sciences : official journal of the European Federation for Pharmaceutical Sciences*. Netherlands, 26(3–4), pp. 295–306. doi: 10.1016/j.ejps.2005.06.010.
17. Woo, S., Krzyzanski, W. and Jusko, W. J. (2007) ‘Target-mediated pharmacokinetic and pharmacodynamic model of recombinant human erythropoietin (rHuEPO)’, *Journal of pharmacokinetics and pharmacodynamics*. United States, 34(6), pp. 849–868. doi: 10.1007/s10928-007-9074-0.
18. Singh, I. , Nagiec EE, Thompson JM, Krzyzanski W, Singh P. A (2015) ‘A Systems Pharmacology Model of Erythropoiesis in Mice Induced by Small Molecule Inhibitor of Prolyl Hydroxylase Enzymes’, *CPT: pharmacometrics & systems pharmacology*. United States, 4(2), p. e12. doi: 10.1002/psp4.12.
19. Nombela-Arrieta, C. and Manz, M. G. (2017) ‘Quantification and three-dimensional microanatomical organization of the bone marrow’, *Blood advances*. American Society of Hematology, 1(6), pp. 407–416. doi: 10.1182/bloodadvances.2016003194.
20. Cheung, W., Minton, N. and Gunawardena, K. (2001) ‘Pharmacokinetics and pharmacodynamics of epoetin alfa once weekly and three times weekly’, *European journal of clinical pharmacology*. Germany, 57(5), pp. 411–418. doi: 10.1007/s002280100324.
21. Provenzano, R., Gracia-Mayol L, Suchinda P, Von Hartitzsch B, Woollen S, Zabaneh R,*et al.* (2004) ‘Once-weekly epoetin alfa for treating the anemia of chronic kidney disease’, *Clinical nephrology*. Germany, 61(6), pp. 392–405. doi: 10.5414/cnp61392.
22. Suranyi, M. G., Lindberg JS, Navarro J, Elias C, Brenner RM, Walker R. (2003) ‘Treatment of anemia with darbepoetin alfa administered de novo once every other week in chronic kidney disease’, *American journal of nephrology*. Switzerland, 23(2), pp. 106–111. doi: 10.1159/000068041
23. Pergola, P. E.,Spinowitz BS, Hartman CS, Maroni BJ, Haase VH. Vadadustat, (2016) ‘Vadadustat, a novel oral HIF stabilizer, provides effective anemia treatment in nondialysis-dependent chronic kidney disease’, *Kidney international*. United States, 90(5), pp. 1115–1122. doi: 10.1016/j.kint.2016.07.019.
24. Holdstock, L., Cizman B, Meadowcroft AM, Biswas N, Johnson BM, Jones D, *et al.* (2018) ‘Daprodustat for anemia: a 24-week, open-label, randomized controlled trial in participants with chronic kidney disease’, *Clinical kidney journal*. England, 12(1), pp. 129–138. doi: 10.1093/ckj/sfy013.
25. Nissenson, A. R.,Swan SK, Lindberg JS, Soroka SD, Beatey R, Wang C, *et al.* (2002) ‘Randomized, controlled trial of darbepoetin alfa for the treatment of anemia in hemodialysis patients’, *American journal of kidney diseases : the official journal of the National Kidney Foundation*. United States, 40(1), pp. 110–118. doi: 10.1053/ajkd.2002.33919.
26. Locatelli, F., Canaud B, Giacardy F, Martin‐Malo A, Baker N, Wilson J. (2003) ‘Treatment of anaemia in dialysis patients with unit dosing of darbepoetin alfa at a reduced dose frequency relative to recombinant human erythropoietin (rHuEpo)’, *Nephrology, dialysis, transplantation : official publication of the European Dialysis and Transplant Association - European Renal Association*. England, 18(2), pp. 362–369. doi: 10.1093/ndt/18.2.362.
27. Haase, V. H., Chertow GM, Block GA, Pergola PE, deGoma EM, Khawaja Z, *et al.* (2019) ‘Effects of vadadustat on hemoglobin concentrations in patients receiving hemodialysis previously treated with erythropoiesis-stimulating agents’, *Nephrology, dialysis, transplantation : official publication of the European Dialysis and Transplant Association - European Renal Association*. England, 34(1), pp. 90–99. doi: 10.1093/ndt/gfy055.
28. Meadowcroft, A. M.,Cizman B, Holdstock L, Biswas N, Johnson BM, Jones D, *et al.* (2018) ‘Daprodustat for anemia: a 24-week, open-label, randomized controlled trial in participants on hemodialysis’, *Clinical kidney journal*. England, 12(1), pp. 139–148. doi: 10.1093/ckj/sfy014.
29. Eden, E.,Geva-Zatorsky N, Issaeva I, Cohen A, Dekel E, Danon T, *et al.* (2011) ‘Proteome half-life dynamics in living human cells’, *Science (New York, N.Y.)*. United States, 331(6018), pp. 764–768. doi: 10.1126/science.1199784.
30. Spencer, S. L. Gaudet S, Albeck JG, Burke JM, Sorger PK. (2009) ‘Non-genetic origins of cell-to-cell variability in TRAIL-induced apoptosis’, *Nature*. England, 459(7245), pp. 428–432. doi: 10.1038/nature08012
31. Sigal, A., Milo R, Cohen A, Geva-Zatorsky N, Klein Y, Liron Y, *et al.* (2006) ‘Variability and memory of protein levels in human cells’, *Nature*. England, 444(7119), pp. 643–646. doi: 10.1038/nature05316.
32. Eckardt, K.-U, .Agarwal R, Aswad A, Awad A, Block GA, Bacci MR,.et al (2021) ‘Safety and Efficacy of Vadadustat for Anemia in Patients Undergoing Dialysis’, The New England journal of medicine. United States, 384(17), pp. 1601–1612. doi: 10.1056/NEJMoa2025956.
